# Adipogenesis in triple-negative breast cancer is associated with unfavorable tumor immune microenvironment and with worse survival

**DOI:** 10.1038/s41598-021-91897-7

**Published:** 2021-06-15

**Authors:** Masanori Oshi, Yoshihisa Tokumaru, Fernando A. Angarita, Lan Lee, Li Yan, Ryusei Matsuyama, Itaru Endo, Kazuaki Takabe

**Affiliations:** 1 Department of Surgical Oncology, Roswell Park Comprehensive Cancer Center, Elm & Carlton Streets, Buffalo, NY 14263 USA; 2 Department of Gastroenterological Surgery, Yokohama City University Graduate School of Medicine, Yokohama, Kanagawa 236-0004 Japan; 3 Department of Surgical Oncology, Graduate School of Medicine, Gifu University, 1-1 Yanagido, Gifu, 501-1194 Japan; 4 Department of Biostatistics & Bioinformatics, Roswell Park Comprehensive Cancer Center, Buffalo, NY 14263 USA; 5 Division of Digestive and General Surgery, Niigata University Graduate School of Medical and Dental Sciences, Niigata, 951-8520 Japan; 6 Department of Breast Surgery, Fukushima Medical University School of Medicine, Fukushima, 960-1295 Japan; 7 Department of Breast Surgery and Oncology, Tokyo Medical University, Tokyo, 160-8402 Japan; 8 Department of Surgery, Jacobs School of Medicine and Biomedical Sciences, State University of New York, Buffalo, NY 14263 USA

**Keywords:** Breast cancer, Lipid signalling, Tumour immunology, Cancer genomics

## Abstract

Cancer-associated adipocytes are known to cause inflammation; however, the role of adipogenesis, the formation of adipocytes, in breast cancer is unclear. We hypothesized that intra-tumoral adipogenesis reflects a different cancer biology than abundance of intra-tumoral adipocytes. The Molecular Signatures Database Hallmark adipogenesis gene set of gene set variant analysis was used to quantify adipogenesis. Total of 5,098 breast cancer patients in multiple cohorts (training; GSE96058 (*n* = 3273), validation; TCGA (*n* = 1069), treatment response; GSE25066 (*n* = 508) and GSE20194 (*n* = 248)) were analyzed. Adipogenesis did not correlate with abundance of adipocytes. Adipogenesis was significantly lower in triple negative breast cancer (TNBC). Elevated adipogenesis was significantly associated with worse survival in TNBC, but not in the other subtypes. High adipogenesis TNBC was significantly associated with low homologous recombination deficiency, but not with mutation load. High adipogenesis TNBC enriched metabolism-related gene sets, but neither of cell proliferation- nor inflammation-related gene sets, which were enriched to adipocytes. High adipogenesis TNBC was infiltrated with low CD8^+^ T cells and high M2 macrophages. Although adipogenesis was not associated with neoadjuvant chemotherapy response, high adipogenesis TNBC was significantly associated with low expression of *PD-L1* and *PD-L2* genes, and immune checkpoint molecules index. In conclusion, adipogenesis in TNBC was associated with cancer metabolism and unfavorable tumor immune microenvironment, which is different from abundance of adipocytes.

## Introduction

Both breast cancer screening and advancement in therapeutics have significantly improved the prognosis of breast cancer patients. Nevertheless, breast cancer remains the most common cause of cancer-related death among women in the US^1^. Obesity, one of the risk factors in several cancer, is related with breast cancer carcinogenesis, cancer progression, and poor clinical outcome^2^. Numerous studies have shown the biological link between obesity and cancer progression. For example, adipose tissue evokes inflammation characterized by adipocytes surrounded by macrophages forming crown-like structures^3^ and increases tissue levels of proinflammatory mediators^4^. We have reported that sphingosine-1-phosphate (S1P) produced in breast cancer cells by sphingosine kinase 1 links obesity, chronic inflammation and metastasis^5^, and that S1P take part in doxorubicin resistance in obesity-related breast cancer^6^.

Intra-tumoral adipocytes, also known as cancer-associated adipocytes, interact with cancer cells and secrete the inflammatory cytokines (e.g.: IL-6 and TNF-α)^7^. These cytokines contribute to pro-cancer inflammation, which is known to aggravate cancer progression, and activate several pathways, such as epithelial-mesenchymal transition (EMT), Notch1 signaling, and angiogenesis^8–11^. We previously reported that intra-tumoral adipocytes are associated with inflammation and metastasis but with less cell proliferation and better patient survival using multiple large breast cancer cohorts with transcriptomes^12^. These data suggest that abundance of intra-tumoral adipocytes inversely reflects the cancer cell density of the bulk tumor thus less aggressive cancer. On the other hand, the clinical relevance of adipogenesis, the generation of adipocytes as opposed to the existence of adipocytes investigated in the previous study, has never been shown in large patient cohorts.

To date, our group has been investigating the role of biological pathways in breast cancer progression and their clinical relevance. We and the others have repeatedly shown that competitive scoring using multiple genes can provide more accurate predictions in capturing biological pathways than single gene expressions since multiple genes are involved in cancer progression^13,14^. Gene set-based approach that utilize multiple genes take the coordination of genes into account, reduce model complexity and increase the explanatory power of prediction^15^. We have used Gene Set Variant Analysis (GSVA) score with Hallmark gene sets in The Molecular Signatures Database hallmark gene set collection, which allows exploration of the biological activity of a signaling pathway of interest^16^. This method has been widely used to score pathway activity from global gene expression data thereby showing the clinical relevance of G2M checkpoint^17^, E2F targets^18^, MYC targets^19^, KRAS signaling up^20^, estrogen response^21^ and angiogenesis pathway^22^ in breast cancer.

Here, we hypothesize that the intra-tumoral adipogenesis activity is associated with different cancer biology from abundance of adipocytes in breast cancer. To test our hypothesis, we defined the adipogenesis activity by GSVA Hallmark adipogenesis gene set and analyzed in total 5,098 breast cancer patients in testing and validation cohorts.

## Materials and methods

### Clinical and transcriptomic data of breast cancer cohors

Clinical information of 1,069 female breast cancer patients in The Cancer Genome Atlas Breast Cancer cohort (TCGA-BRCA) were obtained from Pan-Cancer Clinical Data Resource^23^ and RNA-sequencing data were obtained through cBio Cancer Genomic Portal^24^, we previously reported^25,26^. We also obtained clinical and RNA-sequencing data of GSE96058 cohort (*n* = 3273)^27^ from Gene Expression Omnibus (GEO) repository. We used the studies by Siegel et al. (GSE110590; *n* = 83)^28^ for metastatic breast cancer analysis, and by Symmans et al. (GSE25066; *n* = 508)^29^ and Shi et al. (GSE20194; *n* = 248)^30^ for drug response analysis. Gene expression data were used after transformed for log_2_.

TCGA and GEO data sets, are de-identified publicly available database, Institutional Review Board was waived.

### Gene set variation analysis (GSVA) and gene set enrichment analysis (GSEA)

Intra-tumoral adipogenesis pathway score was measured by Gene Set Variation Analysis (GSVA)^31^ Bioconductor package using “Hallmark_adipogenesis” gene set in the Molecular Signatures Database (MSigDB) collections^16^, similar to how we measured other several signaling score^21,32,33^. In the gene set enrichment analysis (GSEA)^34^, the statistical significance was defined as false discovery rate (FDR) less than 25%, recommended by GSEA software.

### Other score

Immune cells and adipocytes fraction were estimated by xCell algorithm^35^ using R (Table S4). Cytolytic activity score was based on the expression levels of granzyme A (*GZMA*) and perforin 1 (*PF1*), which was reported by Rooney et al.^36^. Homologous recombination deficiency (HRD), fraction altered, silent and non-silent mutation, single nucleotide variant (SNV) and Indel neoantigens scores were calculated by Thorsson et al. in the TCGA^37^. Immune checkpoint molecules index was calculated with mRNA-sequence data of several immune checkpoint molecules, including *ADORA2A (A2AR), CD274 (PD-L1), PDCD1 (PD1), CTLA4, HAVCR2 (TIM3), IDO1, IDO2, PDCD1LG2 (PD-L2), TIGIT, VISTA (C10orf54),* and *VTCN1 (B7-H4),* following the description by Balli et al.^38^.

### Statistical analysis

R software (version 4.0.1) was used for analyses in the study. Median value of the adipogenesis pathway score was utilized as a cut-off to divide two adipogenesis score groups within cohorts. Mann–Whitney U test and Fisher’s test were used to calculate *p* value in group-comparison analysis. Kaplan–Meier curve with log-rank test was used to survival analysis. xCell score was calculated using R software with xCell algorithm^35^, and used to estimate several immune cell fraction in tumor microenvironment, as we previously reported^39–43^. The statistical significance was defined as *p*-value less than 0.05.

## Results

### High adipogenesis score was significantly associated with high expression of adipogenesis-related genes

**Figure 1:**
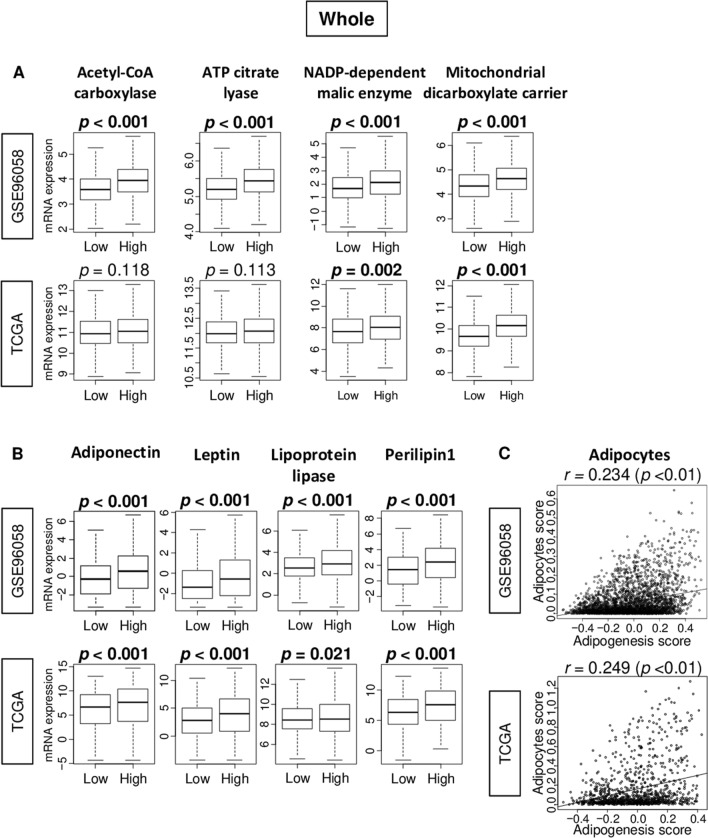
Association of the adipogenesis score with expression of adipogenesis- and adipocyte-related genes, and correlation with adipocyte score in the GSE96058 and TCGA cohorts. Boxplots comparing low and high adipogenesis score tumors of gene expression levels of **(A)** adipogenesis-related genes; Acetyl-CoA carboxylase/*ACLY*, ATP citrate lyase/*ACACA*, NADP-dependent malic enzyme/*SLC25A10*, and mitochondrial dicarboxylate carrier/*ME1*, and **(B)** adipocyte-related genes; adiponectin/*ADIPOQ*, leptin/*LEP*, lipoprotein/*LPL*, and perilipin 1/*PLIN1* in breast cancer. *P*-value was calculated using Mann–Whitney U test. **(C)** Correlation pots between adipogenesis and adipocytes scores. Spearman correlation coefficient (*r*) was used to the analysis.

Adipogenesis pathway score was defined as GSVA score of Hallmark Adipogenesis gene set in the Molecular Signatures Database (MSigDB). The genes included in the score are listed in Supplemental Table S1. Median value was used as a cut-off to divide into low and high adipogenesis score groups within cohorts (Fig. S1). First, in order to examine whether the score reflect adipogenesis in patient breast cancer, we investigated the association of the score with several adipogenesis-related genes (Acetyl-CoA carboxylase/*ACLY*, ATP citrate lyase/*ACACA,* NADP-dependent malic enzyme/*SLC25A10,* and mitochondrial dicarboxylate carrier/*ME1*) and adipocyte-related genes (adiponectin/*ADIPOQ*, leptin/*LEP*, lipoprotein lipase/*LPL*, and perilipin1/*PLIN1*) in the GSE96058 and TCGA cohorts. High adipogenesis score tumors were significantly associated with high expression of all adipogenesis-related genes examined in the GSE96058 cohort (Fig. 1A; all *p* < 0.001). This result was validated by the TCGA cohort, except for Acetyl-CoA carboxylase/*ACLY* and ATP citrate lyase/*ACACA* genes. Whereas high adipogenesis score tumors were significantly associated with high expression of adiponectin/*ADIPOQ*, leptin/*LEP*, lipoprotein lipase/*LPL*, and perilipin1/*PLIN1* genes in both cohorts (Fig. 1B). High adipogenesis score tumors were also significantly associated with high expression of other adipogenesis-related genes, including *CEBPPA, AP2 (FABP4),* and *GLUT4 (SLC2A4)*, and low expression of PPAR (*PPARA*), in both cohort (Fig. S2, all *p* < 0.001). On the contraly, there was no correlation between adipogenesis and adipocyte scores in neither of the cohorts (Fig. 1C; spearman rank correlation (*r*) = 0.234, and 0.249, respectively). The adipogenesis score also not correlated with leptin (*LEP*) to adiponectin (*ADIPOQ*) ratios in the GSE96058 cohort (Fig. S3, *r* = 0.063, *p* < 0.01). These results suggest that the score reflects adipogenesis but does not overlap with adipocyte score.

### Adipogenesis score was low in triple-negative (TNBC) and basal subtype breast cancer, although not associated with clinical aggressiveness

**Figure 2:**
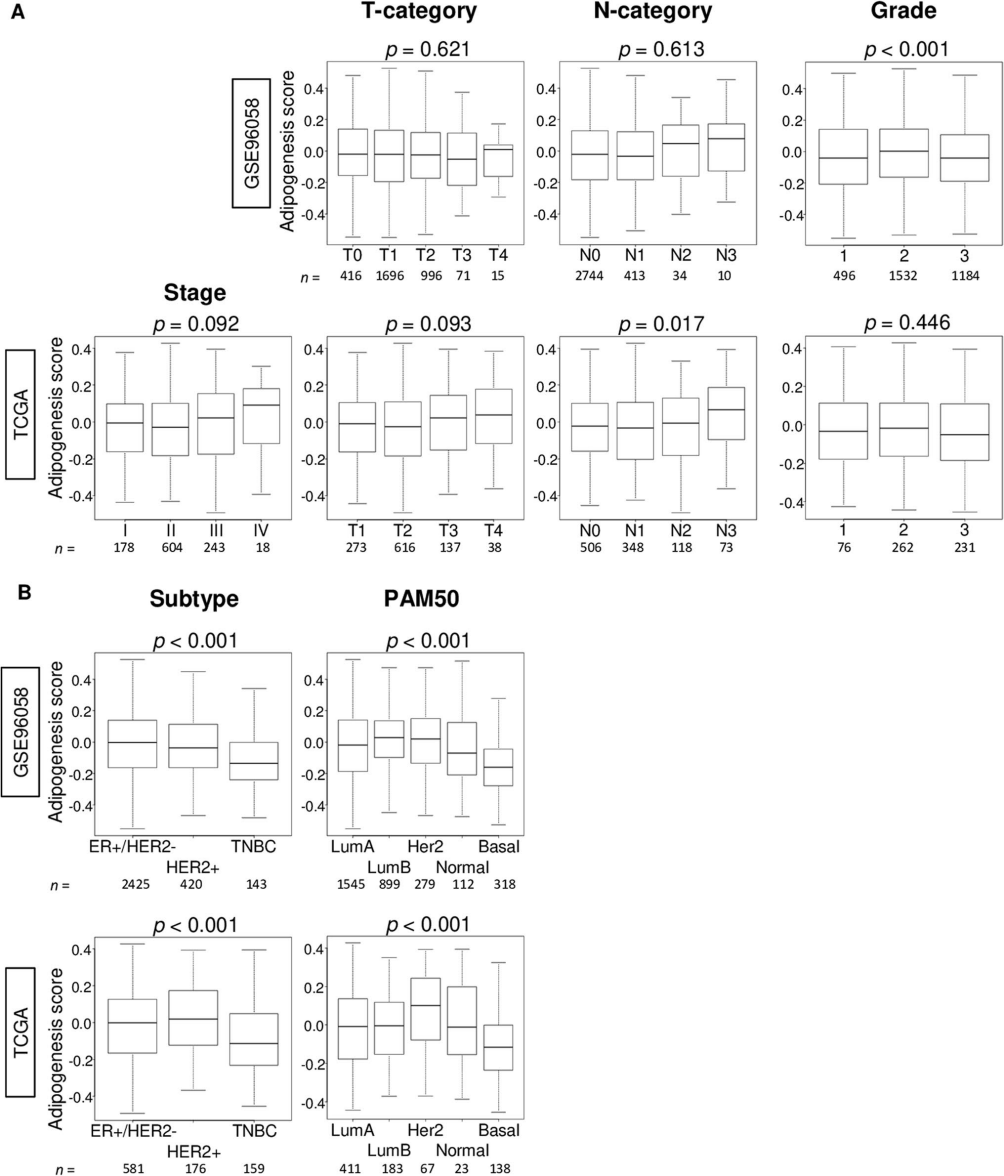
Association of the adipogenesis pathway score with clinical features in the GSE96058 and TCGA cohorts. Boxplots of the adipogenesis scores of tumors by **(A)** American Joint Committee on Cancer stage, tumor size (T-category) and lymph node positivity (N-category), Nottingham pathological grade, and **(B)** breast cancer subtype, and Pam50 classifications. *P*-value was calculated using Kruskal–Wallis test. Group sizes are shown underneath the plots.

To investigate the association of the adipogenesis score and clinical features of breast cancer, we examined American Joint Committee on Cancer (AJCC) cancer staging, T-category, N-category, Nottingham pathological grade, subtype, and Pam50 in both the GSE96058 and TCGA cohorts. The score was not associated with aggressive clinical parameters including AJCC stage, and T-category in both cohort (Fig. 2A). High score was significantly associated with high N-category (Fig. 2A; *p* = 0.017) only in TCGA cohort and not in GSE96058 cohort (Fig. 2A; *p* = 0.613). Although the mean value of adipogenesis score appeared high in Stage IV, it did not reach statistically significant difference probably due to its small sample size (18/1069 (1.6%) of the TCGA). Adipogenesis score was significantly higher in the Nottingham histological grade 2 compared from the other grade in the GSE96058, but not in the TCGA cohort. Adipogenesis score was significantly low in triple-negative breast cancer (TNBC) defined by immunohistochemistry (IHC), and basal type defined by Pam50 classification in both cohorts (Fig. 2B; all *p* < 0.001). Clinical and pathological features of low and high adipogenesis score in the GSE96058 (*n* = 3273) and TCGA (*n* = 1069) cohorts are summarized in Supplemental Tables S2 and S3. In addition, we compared the adipogenesis score between the primary and metastatic breast cancer using GSE110590 cohort (Supplementary Fig. S4). We found that although there was no significant difference overall, the score was significantly high in Liver metastasis compared from the primary breast cancer (*p = 0.025). These results suggested that the adipogenesis score was not associated with clinical cancer aggressiveness, but TNBC has significantly less intra-tumoral adipogenesis.

### High adipogenesis score is significantly associated with worse survival in TNBC, but not in the other subtypes

**Figure 3:**
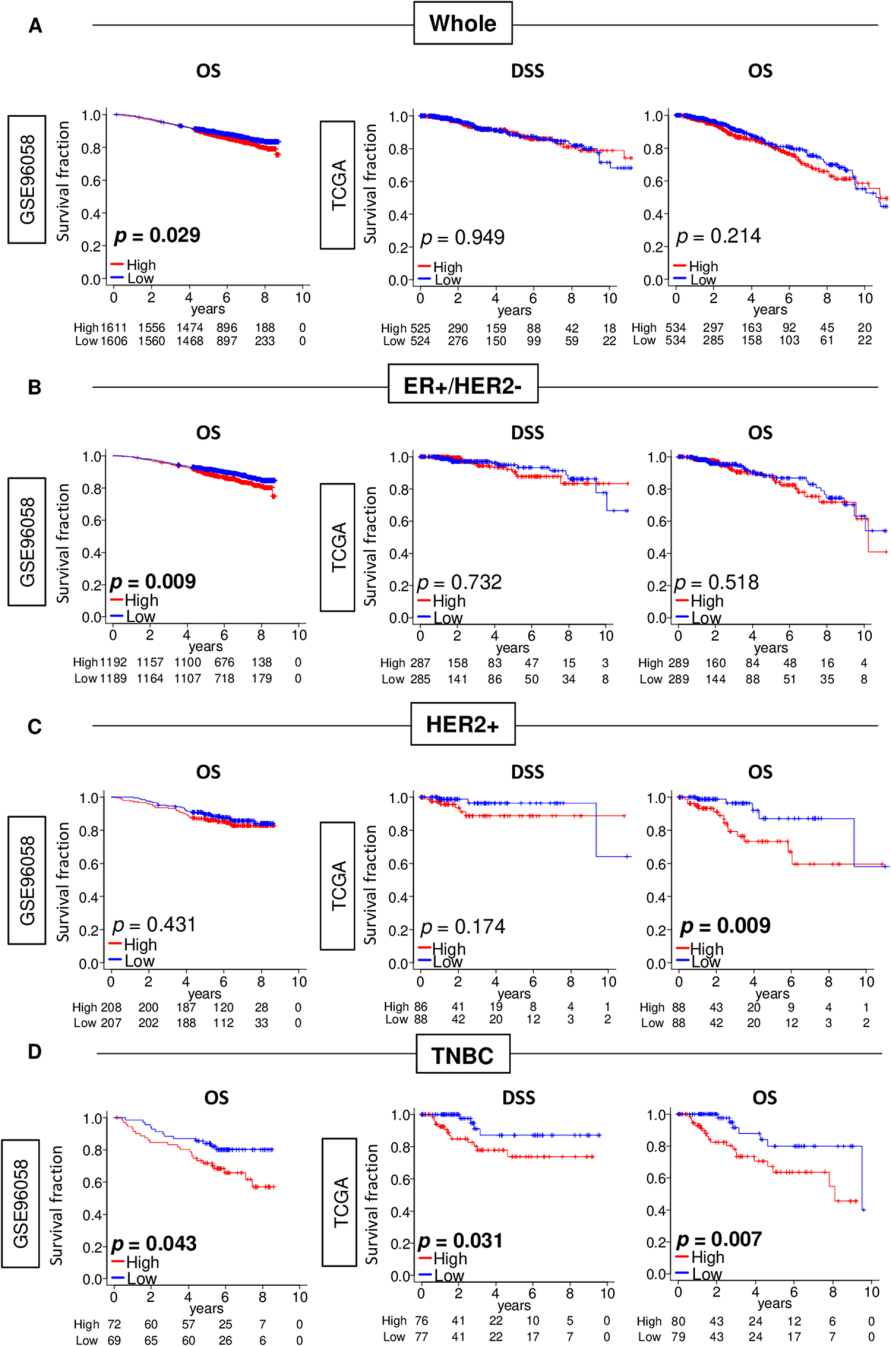
Association of adipogenesis pathway score with patient survival by whole and each subtype in the GSE96058 and TCGA cohorts. Kaplan–Meier plots of Disease-specific survival (DSS) in the TCGA cohort, and overall survival (OS) in both cohorts by adipogenesis low (blue) and high (red) score with **(A)** whole, **(B)** Estrogen receptor (ER)+/HER2−, **(C)** human epidermal growth factor receptor 2 (HER2)-positive, or **(D)** Triple negative breast cancer (TNBC) patients. Median value was used as the cut-off to divide into low and high adipogenesis score groups within each cohort. *P*-value was calculated using log-rank test.

Given that adipogenesis score was significantly different by subtypes, we hypothesized that each subtype may have different relationship with adipogenesis. To test this, we analyzed the disease specific survival (DSS) in the TCGA cohort, and overall survival (OS) in both GES96058 and TCGA cohorts. High adipogenesis score was significantly associated with worse OS of whole and ER-positive/HER2-negative breast cancer patient in the GSE96085 cohort (Fig. 3A), but not in the TCGA cohort (Fig. 3A). Furthermore, high adipogenesis score was significantly associated with worse OS in HER2-positive breast cancer in the TCGA, but not validated in the GSE96058 cohort. Of note, whether the patients received HER2 targeted therapy or not were unclear on the GSE96058 and TCGA cohorts, which may significantly confound the data and its interpretation. On the other hand, in TNBC, high score was significantly associated with worse OS in the GSE96058 (Fig. 3C; *p* = 0.007), and worse DSS and OS in the TCGA (Fig. 3C; *p* = 0.031, and *p* = 0.007). These results suggest that high adipogenesis score was associated with worse prognosis particularly in TNBC patients.

### High adipogenesis score in TNBC significantly enriched metabolism-related gene sets, but not inflammation nor cell proliferation-related gene sets

**Figure 4:**
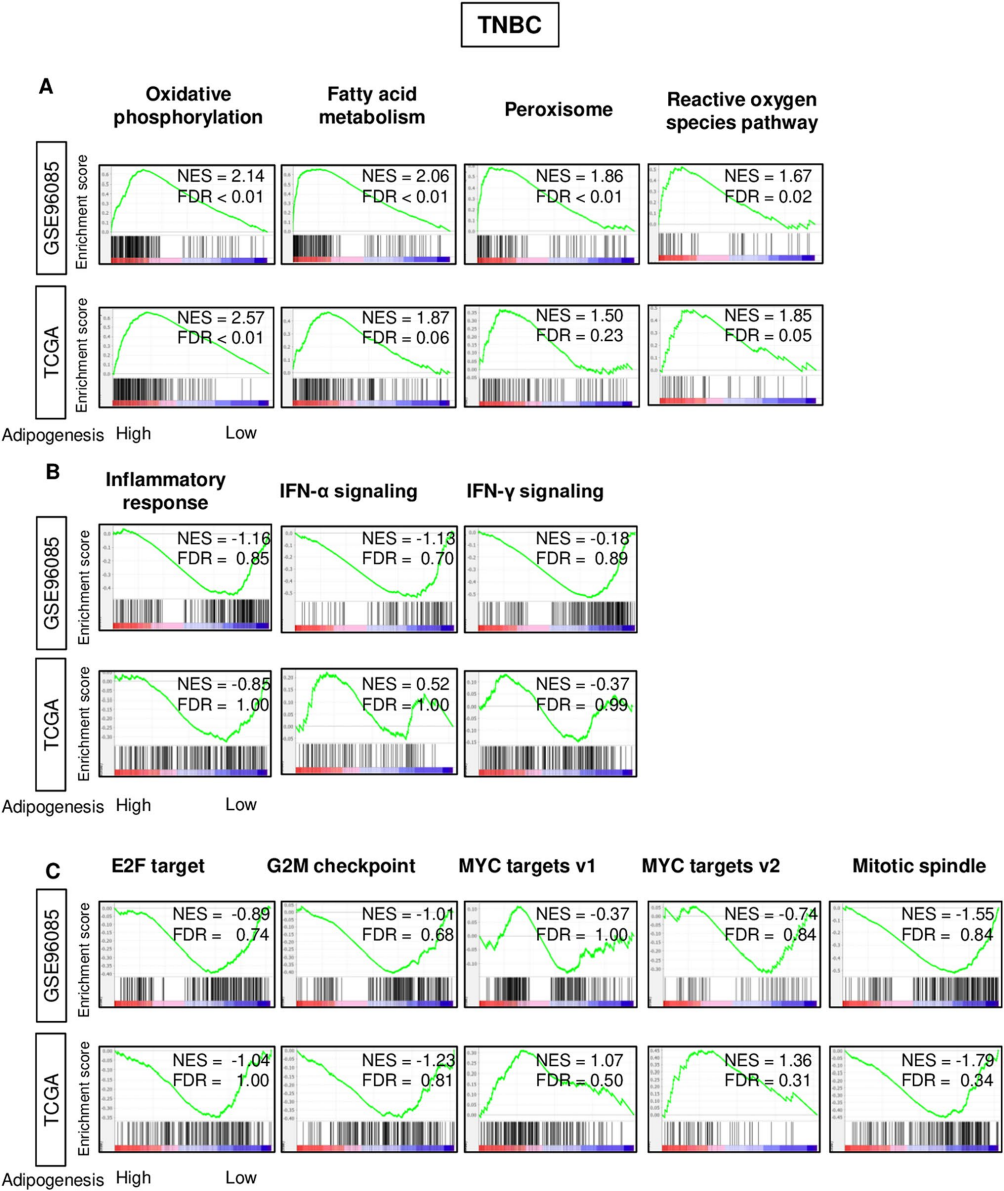
Gene Set Enrichment Assay (GSEA) on adipogenesis score in TNBC of both GSE96058 and TCGA cohorts. Gene set enrichment plots of **(A)** Hallmark metabolism-related gene sets (oxidative phosphorylation, fatty acid metabolism, peroxisome, reactive oxygen species pathway) **(B)** inflammation-related gene sets (inflammatory response, interferon (IFN)-α response and IFN-γ response) and (**C**) cell proliferation-related gene sets (E2F target, G2M checkpoint, mitotic spindle, and MYC target v1 and v2) in both cohorts with normalized enrichment score (*NES*) and false discovery rate (*FDR*).

Given that the adipogenesis score was significantly different only in TNBC, we have focused our investigation on TNBC. In order to elucidate the underlying mechanism of the survival difference by the score in TNBC, we performed the Gene set enrichment analysis (GSEA) of Hallmark gene sets between high and low adipogenesis score groups in both TCGA and GSE96058 cohorts. High adipogenesis score significantly enriched metabolism-related gene sets; oxidative phosphorylation, fatty acid metabolism, and cholesterol homeostasis gene sets, as well as peroxisome and reactive oxygen species pathway in TNBC of both cohorts (Fig. 4A). Surprisingly, there was no significant enrichment of none of the inflammation-related (inflammatory response, interferon-alpha signaling, interferon-gamma signaling) nor cell proliferation-related (E2F targets, G2M checkpoint, MYC targets V1 and V2, mitotic spindle) gene sets, which were all enriched to low adipocyte score in our previous publication^12^ (Fig. 4B,C).

### High adipogenesis TNBC was significantly associated with low homologous recombination deficiency (HRD) and low fraction of CD8^+^ T cells

**Figure 5:**
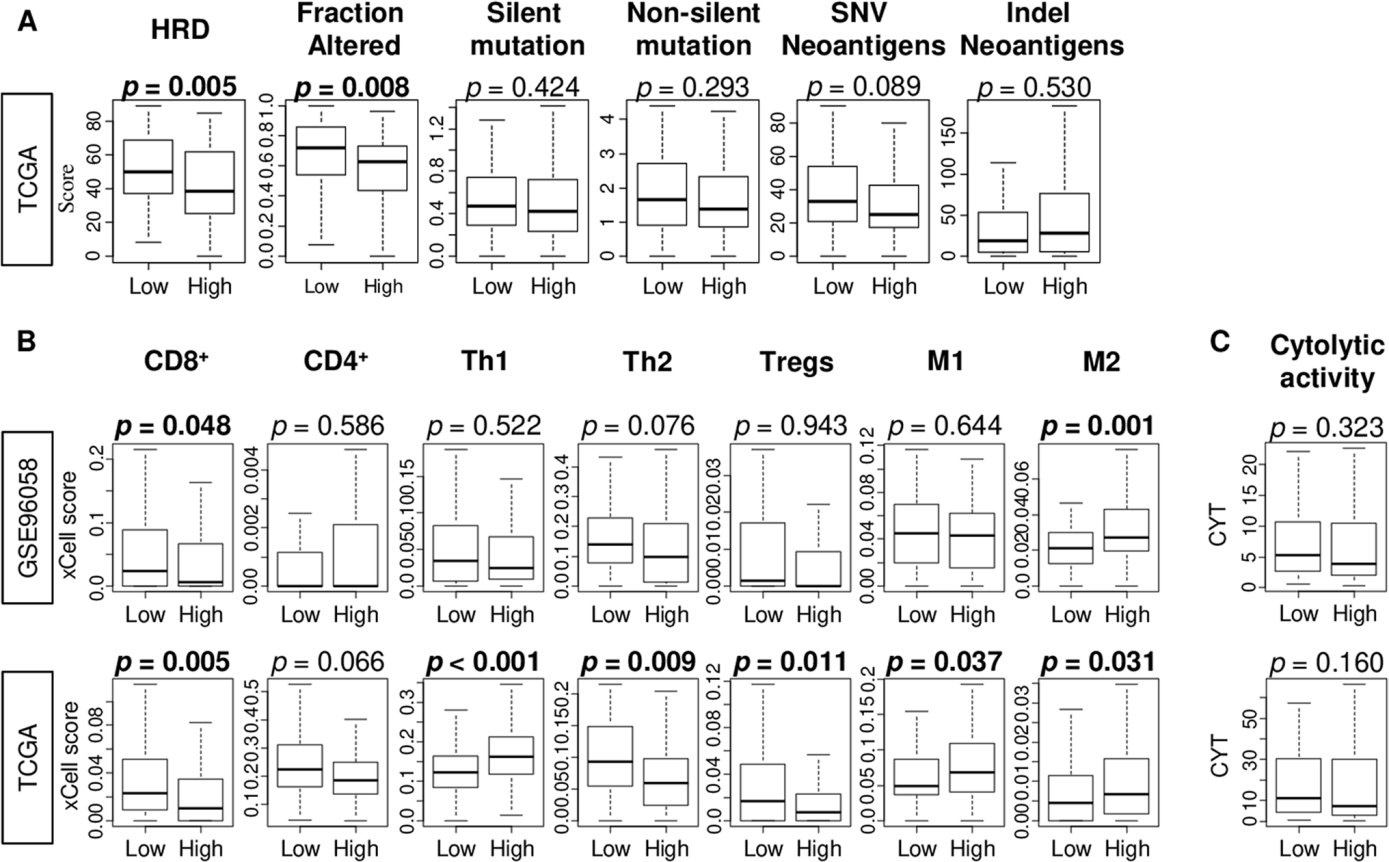
Homologous recombination deficiency (HRD), mutation load, fractions of tumor infiltrating immune cells and cytolytic activity between low and high adipogenesis triple negative breast cancer (TNBC) in the GSE96058 and TCGA cohorts. **(A)** Boxplots of HRD, altered fraction, silent and non-silent mutation, single nucleotide variation (SNV) and indel neoantigens by the adipogenesis in TNBC. **(B)** The comparison of immune cells; CD8^+^ T cells, CD4^+^ memory T cells, T helper type 1 cells (Th1) and Th2, regulatory T cells (Tregs), M1 and M2 macrophage, and **(C)** cytolytic activity score (CYT). Median value was used as the cut-off to divide into low and high adipogenesis score groups within each cohort. *P*-value was calculated using Mann–Whitney U test.

Given the difference between adipogenesis and abundance of adipocytes in GSEA, we further investigated the relationship between adipogenesis and infiltrating immune cells in TNBC. Homologous recombination deficiency (HRD) is one of the mechanisms that generate mutaions and high mutation load lead to generation of neoantigens that is thought to attract immune cells to tumor microenvironment^44^. We found that high adipogenesis was significantly associated with low level of HRD and amount of fraction altered (Fig. 5A; *p* = 0.005, *p* = 0.008), but not with neither silent or non-silent mutation, nor single nucleotide variation (SNV) and indel neoantigens in TNBC (Fig. 5A; *p* = 0.008, 0.424. 0.293, 0.089, and 0.530, respectively) in TNBC. On the other hand, we found that a high adipogenesis TNBC was significantly associated with low fraction of CD8^+^ T cells and high fraction of M2 macrophages consistently in both cohorts (Fig. 5B). High adipogenesis was significantly associated with low fraction of Th2 and Tregs, and high fraction of Th1 and M1 macrophages in the TCGA cohort (Fig. 5B) but not in GSE96058. Cytolytic activity score that reflect the overall immune response was not associated with adipogenesis (Fig. 5C). These findings suggest that high adipogenesis TNBC have less HRD and low infilatration of CD8^+^ T cells, but high infiltration of M2 macrophages.

### High adipogenesis was not associated with neoadjuvant chemotherapy (NAC) response, but significantly associated with low expression of PD-L1 and PD-L2, and inhibitory checkpoint index

**Figure 6:**
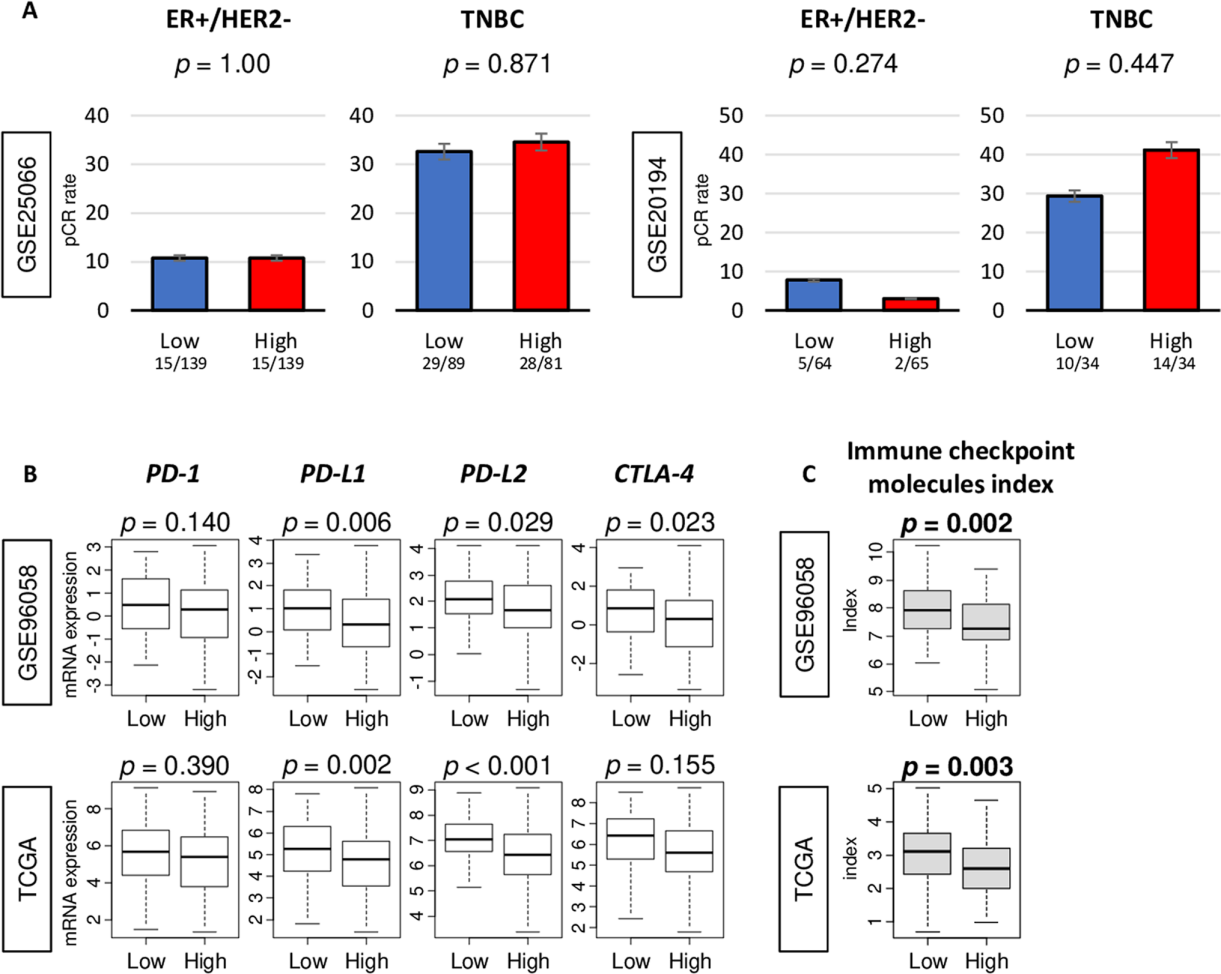
Association of adipogenesis score with neoadjuvant chemotherapy (NAC) response and expression of immune checkpoint molecules in TNBC. **(A)** Bar plots of pathological compete response (pCR) rate after NAC between low (blue) and high (red) adipogenesis score in ER+/HER2− and TNBC in the GSE25066 and GSE20194 cohorts. The numbers under the chart are pCR case number/number of patients in that group that makes the pCR rate. Error bar showed Standard Error. *P*-value was calculated by Fisher’s test. **(B)** Boxplots of comparison between low- and high-adipogenesis score with mRNA expression of PD-1/*PDCD1*, PD-L1/*CD274*, PD-L2/*PDCD1LG2*, and *CTLA4* genes, and **(C)** immune checkpoint molecules index in TNBC in the GSE96058 and TCGA cohorts. *P*-value was calculated using Mann–Whitney U test. Median value was used as the cut-off to divide into low- and high- adipogenesis score groups within each cohort. *PD-1* programmed cell death 1; *PD-L1* programmed cell death 1 ligand 1, *PD-L2* programmed cell death 1 ligand 2, *CTLA4* cytotoxic T-lymphocyte-associated protein 4.

Breast cancer with high infiltrating CD8^+^ T cells are known to have better response to NAC, especially in TNBC^45^. Since high adipogenesis was associated with low CD8^+^ T cells, we expected that high adipogenesis is associated with less achievement of pathological compete response (pCR) after NAC. Contrary to our expectation, there was no difference in pCR rate between low and high adipogenesis score in ER-positive/HER2-negative nor TNBC subtype neither in the GSE25066 or GSE20194 cohorts (Fig. 6A). On the other hand, high adipogenesis TNBC was significantly associated with low expression of PD-L1 and PD-L2, which are major immune checkpoint molecules in both GSE96058 and TCGA cohorts (Fig. 6B). Further, high adipogenesis breast cancer, was significantly associated with low level of immune checkpoint molecules index in TNBC as well as in whole cohort of both GSE96058 and TCGA (Figs. 6C and S5). These findings suggest that adipogenesis is not associated with NAC response, but high adipogenesis is associated with low levels of immune checkpoint molecules, especially *PD-L1* and *PD-L2*.

## Discussion

In this study, we demonstrated the clinical relevance of intra-tumoral adipogenesis in breast cancer using a score defined by transcriptome. The adipogenesis score was significantly associated with expression of adipogenesis- and adipocyte-related genes but did not correlate with fraction of adipocytes in breast cancer. Adipogenesis score was significantly low in TNBC and basal type among the other subtypes. High adipogenesis was significantly associated with worse survival, particularly in TNBC. High adipogenesis TNBC enriched several metabolism-related gene sets; oxidative phosphorylation, fatty acid metabolism, peroxisome, and reactive oxygen species pathway, but none of cell proliferation-related nor inflammation-related gene sets were enriched, unlike adipocyte infiltration. High adipogenesis TNBC was significantly associated with low HRD and altered fraction, and less CD8^+^ T cells and high M2 macrophage infiltrations. Although there was no significant difference in achievement of pCR after NAC by adipogenesis neither in ER-positive/HER2-negative or TNBC, high adipogenesis TNBC was significantly associated with low expression of *PD-L1* and *PD-L2* and with low immune checkpoint molecules index.

Intra-tumoral adipocytes are known to cause inflammation, hypoxia, and angiogenesis^8–10^, which are associated with poor patient survival. It is known that adipocytes participate in a highly complex vicious cycle orchestrated by cancer cells to promote cancer progression^46^. We previously demonstrated that high fraction of intra-tumoral adipocyte enriched of inflammation-related and metastasis-related gene sets, and less enriched of cell proliferation-related gene sets in gene set enrichment analyses; however, it was not associated with survival^12^. We speculated that this is because highly proliferative cancer develops cell density too high for adipocytes to exist in such tumor microenvironment. Thus, aggressive biology evoked by intra-tumoral adipocytes are counterbalanced by selection of less proliferative cancer that resulted in no difference in clinical outcome by the abundance of adipocytes. Although adipocytes are the final product of adipogenesis, we found that correlation between adipogenesis and adipocytes was very weak in breast cancer, and high adipogenesis TNBC did not enrich similar gene sets as high adipocytes, including immune- and proliferation-related pathways. On the other hand, high adipogenesis TNBC was significantly associated with metabolism-related gene sets, but not with the abundance of adipocytes. These results led us to speculate that TNBC with high metabolic activity including adipogenesis is associated with n poor survival due to high infiltration of unfavorable immune cells rather than aggressive cancer biology such as high cell proliferation. Our findings demonstrate that adipogenesis score better reflects the fat-related signaling pathway rather than abundance of adipocytes. The leptin to adiponectin ratio in serum is an important indicator of cancer risk^47,48^. Although there was no evidence of it in tumor tissue, the adipogenesis score was not associated with leptin to adiponectin ratio in this study. It is well known that TNBC is a heterogeneous subtype, and recently studies have categorized TNBC into several groups (Burstein et al.^49^, Lehmann et al.^50^)). We showed that not only TNBC, which was categorized by IHC analysis, but also basal type, categorized by PAM50, was significantly associated with lower level of adipogenesis score compared to other subtypes. To this end it will be interesting to investigate the association of these four new categories of TNBC reported by Burstein et al.^49^ with the adipogenesis score; however, we are unable to do so given that we do not have access to the data on these new categorizations of TNBC in the study cohorts.

Although abundance of adipocytes in TNBC did not associated with infiltration of CD8^+^ T cells and macrophages, high adipogenesis TNBC was significantly associated with less CD8^+^ T cell and high M2 macrophage infiltration and poor survival. These findings suggest that adipogenesis activity and abundance of adipocytes reflect different biology in breast cancer. They indicate that the clinical relevance of intra-tumoral adipogenesis may not be due to cell cycle or inflammation, but due to less favorable and more unfavorable tumor immune microenvironment. It is well known that TNBC has higher infiltrations of immune cells among breast cancer subtype^45^. Our group as well as others demonstrated that TNBCs with high CD8^+^ T cell infiltration have better survival, and significantly associated with high expression of immune checkpoint molecules^39^. Macrophages participate in the entire tumor progression process, as an important subset of tumor infiltrating immune cells, also known as tumor-associated macrophages. M2 macrophages are recognized as pro-cancer immune cells that inhibit antigen presenting function, thus promote immune escape. Relationship between tumor-associated macrophages and cancer-associated adipocytes are well described in breast cancer^11^. Crown-like structure macrophages that surround dead or dying adipocytes are often found in adipose tissue^51^. These are a histologic hallmark of the proinflammatory process which adipose tissue contributes to the increased risk and worse prognosis of breast cancer in obesity^52^. The number and density of crown-like structure macrophages increase in proportion to the size and number of adipocytes and the abundance of macrophages in breast cancer. Crown-like structure macrophages release fatty acids and triglycerides, which are two of the most common metabolites of intra-tumoral adipocytes that release inflammatory factors and cytokines that promote cancer progression^53^.

Recently there has been increased interest in immune checkpoint molecules due to the success of immunotherapy^54^. *PD-L1* is a pro-tumorigenic immune checkpoint molecule that facilitates a tumor’s ability to escape from the host’s immune system. Anti-*PD-L1* therapy is now a standard of care for the management of many types of cancer including TNBC. Wu et al. reported that adipogenic differentiation and *PD-L1* expression of adipocytes are tightly associated with therapeutic efficacy of immune checkpoint inhibition^55^. Wallace et al. demonstrated that inflammation enhanced macrophage infiltration into the adipose tissue and adipogenesis in obesity using in vitro system^56^. Our findings that high adipogenesis was associated with low infiltration of CD8^+^ T cells and M2 macrophages in patients, are in agreement with these mechanisms demonstrated previously using in vivo and in vitro systems. We cannot help but speculate that adipogenesis score may have a utility as predicative biomarker for not only patient survival but also response to immune checkpoint inhibitors for TNBC.

Our study has several limitations. First, we defined adipogenesis by the transcriptomic profile determined by GSEA Hallmark gene set, which may or may not include all the genes that is associated with adipogenesis. Second, this is a retrospective study that utilized multiple large cohorts with robust clinical and transcriptomic data; however, some clinical data such as co-morbidities and therapeutic interventions are missing. The cohorts we analyzed lack details on which patient received what systemic treatment and it is assumed that all the patients underwent “Standard of Care”. This is particularly relevant in HER2-positive breast cancer since its targeted therapy is so dramatically effective despite its known aggressiveness, whether or not the patient underwent the therapy can be a significant confounder of the survival analysis. Another limitation is that our results are based on the analyses of tumor gene expression alone without any proof of mechanistic role of intra-tumoral adipogenesis activity. The currently study did not conduct IHC analysis for tumor immune microenvironment because we did not have access to pathology sections of the tumors in the GSE96058 and TCGA cohorts. On the other hand, the novelty of our study is to assess adipogenesis and tumor immune microenvironment using a score that combines multiple genes instead of couple. Attempt to confirm our findings with 200 stains per tumor for thousands of samples are simply unrealistic. Finally, in order to prove the utility of adipogenesis score as a predictive biomarker, prospective clinical trial is needed.

In conclusion, Intra-tumoral adipogenesis activity did not reflect the similar cancer biology as abundance of adipocytes, but was associated with cancer metabolism and less CD8^+^ T cells and high M2 macrophage infiltration. The score may predict patient survival and response to immune checkpoint inhibitors.

## Supplementary Information

Supplementary Information.
