# Supplementary material for: Adipogenesis in triple-negative breast cancer is associated with unfavorable tumor immune microenvironment and with worse survival
Source: Sci Rep. 2021 Jun 15;11:12541. doi: 10.1038/s41598-021-91897-7 (PMC8206113; doi:10.1038/s41598-021-91897-7)
Supplement: Supplementary file 1 — Supplementary Information. [file 41598_2021_91897_MOESM1_ESM.docx]

SUPPLEMENTAL MATERIALS

**Adipogenesis in Triple-Negative Breast Cancer is Associated with Unfavorable Tumor Immune Microenvironment and with Worse Survival**

Masanori Oshi ^1,2^, Yoshihisa Tokumaru ^1,3^, Fernando A Angarita ^1^, Lan Lee ^1,8^, Li Yan ^4^, Ryusei Matsuyama ^2^, Itaru Endo ^2^ and Kazuaki Takabe ^1,2,5,6,7,8,^*

1: Department of Surgical Oncology, Roswell Park Comprehensive Cancer Center, Buffalo, New York, NY 14263, USA.

2: Department of Gastroenterological Surgery, Yokohama City University Graduate School of Medicine, Yokohama, Kanagawa 236-0004, Japan.

3: Department of Surgical Oncology, Graduate School of Medicine, Gifu University, 1-1 Yanagido, Gifu 501-1194, Japan.

4: Department of Biostatistics & Bioinformatics, Roswell Park Comprehensive Cancer Center, Buffalo, New York, NY 14263, USA.

5: Division of Digestive and General Surgery, Niigata University Graduate School of Medical and Dental Sciences, Niigata 951-8520, Japan.

6: Department of Breast Surgery, Fukushima Medical University School of Medicine, Fukushima 960-1295, Japan.

7: Department of Breast Surgery and Oncology, Tokyo Medical University, Tokyo 160-8402, Japan.

8: Department of Surgery, Jacobs School of Medicine and Biomedical Sciences, State University of New York, Buffalo, New York, NY 14263, USA.

**Table S1. Member genes of the Adipogenesis pathway score.**

| **Original Member** | **Gene Description** |
| --- | --- |
| ABCA1 | ATP binding cassette subfamily A member 1 |
| ABCB8 | ATP binding cassette subfamily B member 8 |
| ACAA2 | acetyl-CoA acyltransferase 2 |
| ACADL | acyl-CoA dehydrogenase long chain |
| ACADM | acyl-CoA dehydrogenase medium chain |
| ACADS | acyl-CoA dehydrogenase short chain |
| ACLY | ATP citrate lyase |
| ACO2 | aconitase 2 |
| ACOX1 | acyl-CoA oxidase 1 |
| ADCY6 | adenylate cyclase 6 |
| ADIG | adipogenin |
| ADIPOQ | adiponectin, C1Q and collagen domain containing |
| ADIPOR2 | adiponectin receptor 2 |
| AGPAT3 | 1-acylglycerol-3-phosphate O-acyltransferase 3 |
| AGPAT6 | glycerol-3-phosphate acyltransferase 4 |
| AIFM1 | apoptosis inducing factor mitochondria associated 1 |
| AK2 | adenylate kinase 2 |
| ALDH2 | aldehyde dehydrogenase 2 family member |
| ALDOA | aldolase, fructose-bisphosphate A |
| ANGPT1 | angiopoietin 1 |
| ANGPTL4 | angiopoietin like 4 |
| ANKRD57 | sosondowah ankyrin repeat domain family member C |
| APLP2 | amyloid beta precursor like protein 2 |
| APOE | apolipoprotein E |
| ARAF | A-Raf proto-oncogene, serine/threonine kinase |
| ARL4A | ADP ribosylation factor like GTPase 4A |
| ATL2 | atlastin GTPase 2 |
| ATP1B3 | ATPase Na+/K+ transporting subunit beta 3 |
| ATP5O | ATP synthase peripheral stalk subunit OSCP |
| BAZ2A | bromodomain adjacent to zinc finger domain 2A |
| BCKDHA | branched chain keto acid dehydrogenase E1 subunit alpha |
| BCL2L13 | BCL2 like 13 |
| BCL6 | BCL6 transcription repressor |
| C3 | complement C3 |
| CAT | catalase |
| CCNG2 | cyclin G2 |
| CD151 | CD151 molecule (Raph blood group) |
| CD302 | CD302 molecule |
| CD36 | CD36 molecule |
| CDKN2C | cyclin dependent kinase inhibitor 2C |
| CHCHD10 | coiled-coil-helix-coiled-coil-helix domain containing 10 |
| CHUK | component of inhibitor of nuclear factor kappa B kinase complex |
| CIDEA | cell death inducing DFFA like effector a |
| CMBL | carboxymethylenebutenolidase homolog |
| CMPK1 | cytidine/uridine monophosphate kinase 1 |
| COL15A1 | collagen type XV alpha 1 chain |
| COL4A1 | collagen type IV alpha 1 chain |
| COQ3 | coenzyme Q3, methyltransferase |
| COQ5 | coenzyme Q5, methyltransferase |
| COQ9 | coenzyme Q9 |
| COX6A1 | cytochrome c oxidase subunit 6A1 |
| COX7B | cytochrome c oxidase subunit 7B |
| COX8A | cytochrome c oxidase subunit 8A |
| CPT2 | carnitine palmitoyltransferase 2 |
| CRAT | carnitine O-acetyltransferase |
| CS | citrate synthase |
| CYC1 | cytochrome c1 |
| CYP4B1 | cytochrome P450 family 4 subfamily B member 1 |
| DBT | dihydrolipoamide branched chain transacylase E2 |
| DDT | D-dopachrome tautomerase |
| DECR1 | 2,4-dienoyl-CoA reductase 1 |
| DGAT1 | diacylglycerol O-acyltransferase 1 |
| DHCR7 | 7-dehydrocholesterol reductase |
| DHRS7 | dehydrogenase/reductase 7 |
| DHRS7B | dehydrogenase/reductase 7B |
| DLAT | dihydrolipoamide S-acetyltransferase |
| DLD | dihydrolipoamide dehydrogenase |
| DNAJB9 | DnaJ heat shock protein family (Hsp40) member B9 |
| DNAJC15 | DnaJ heat shock protein family (Hsp40) member C15 |
| DRAM2 | DNA damage regulated autophagy modulator 2 |
| ECH1 | enoyl-CoA hydratase 1 |
| ECHS1 | enoyl-CoA hydratase, short chain 1 |
| ELMOD3 | ELMO domain containing 3 |
| ELOVL6 | ELOVL fatty acid elongase 6 |
| ENPP2 | ectonucleotide pyrophosphatase/phosphodiesterase 2 |
| EPHX2 | epoxide hydrolase 2 |
| ESRRA | estrogen related receptor alpha |
| ESYT1 | extended synaptotagmin 1 |
| ETFB | electron transfer flavoprotein subunit beta |
| FABP4 | fatty acid binding protein 4 |
| FAH | fumarylacetoacetate hydrolase |
| FAM73B | mitoguardin 2 |
| FAM82A2 | regulator of microtubule dynamics 3 |
| FZD4 | frizzled class receptor 4 |
| G3BP2 | G3BP stress granule assembly factor 2 |
| GADD45A | growth arrest and DNA damage inducible alpha |
| GBE1 | 1,4-alpha-glucan branching enzyme 1 |
| GHITM | growth hormone inducible transmembrane protein |
| GPAM | glycerol-3-phosphate acyltransferase, mitochondrial |
| GPD2 | glycerol-3-phosphate dehydrogenase 2 |
| GPHN | gephyrin |
| GPX3 | glutathione peroxidase 3 |
| GPX4 | glutathione peroxidase 4 |
| GRPEL1 | GrpE like 1, mitochondrial |
| HADH | hydroxyacyl-CoA dehydrogenase |
| HIBCH | 3-hydroxyisobutyryl-CoA hydrolase |
| HSPB8 | heat shock protein family B (small) member 8 |
| IDH1 | isocitrate dehydrogenase (NADP(+)) 1 |
| IDH3A | isocitrate dehydrogenase (NAD(+)) 3 catalytic subunit alpha |
| IDH3G | isocitrate dehydrogenase (NAD(+)) 3 non-catalytic subunit gamma |
| IFNGR1 | interferon gamma receptor 1 |
| IMMT | inner membrane mitochondrial protein |
| ITGA7 | integrin subunit alpha 7 |
| ITIH5 | inter-alpha-trypsin inhibitor heavy chain 5 |
| ITSN1 | intersectin 1 |
| JAGN1 | jagunal homolog 1 |
| LAMA4 | laminin subunit alpha 4 |
| LEP | leptin |
| LIFR | LIF receptor subunit alpha |
| LIPE | lipase E, hormone sensitive type |
| LPCAT3 | lysophosphatidylcholine acyltransferase 3 |
| LPL | lipoprotein lipase |
| LTC4S | leukotriene C4 synthase |
| MAP4K3 | mitogen-activated protein kinase kinase kinase kinase 3 |
| MCCC1 | methylcrotonoyl-CoA carboxylase 1 |
| MDH2 | malate dehydrogenase 2 |
| ME1 | malic enzyme 1 |
| MGLL | monoglyceride lipase |
| MGST3 | microsomal glutathione S-transferase 3 |
| MOSC2 | mitochondrial amidoxime reducing component 2 |
| MRAP | melanocortin 2 receptor accessory protein |
| MRPL15 | mitochondrial ribosomal protein L15 |
| MTCH2 | mitochondrial carrier 2 |
| MYLK | myosin light chain kinase |
| NDUFA5 | NADH:ubiquinone oxidoreductase subunit A5 |
| NDUFAB1 | NADH:ubiquinone oxidoreductase subunit AB1 |
| NDUFB7 | NADH:ubiquinone oxidoreductase subunit B7 |
| NDUFS3 | NADH:ubiquinone oxidoreductase core subunit S3 |
| NKIRAS1 | NFKB inhibitor interacting Ras like 1 |
| NMT1 | N-myristoyltransferase 1 |
| OBFC2A | nucleic acid binding protein 1 |
| OMD | osteomodulin |
| ORM1 | orosomucoid 1 |
| PDCD4 | programmed cell death 4 |
| PEMT | phosphatidylethanolamine N-methyltransferase |
| PEX14 | peroxisomal biogenesis factor 14 |
| PFKFB3 | 6-phosphofructo-2-kinase/fructose-2,6-biphosphatase 3 |
| PFKL | phosphofructokinase, liver type |
| PGM1 | phosphoglucomutase 1 |
| PHLDB1 | pleckstrin homology like domain family B member 1 |
| PHYH | phytanoyl-CoA 2-hydroxylase |
| PIM3 | Pim-3 proto-oncogene, serine/threonine kinase |
| PLIN2 | perilipin 2 |
| POR | cytochrome p450 oxidoreductase |
| PPARG | peroxisome proliferator activated receptor gamma |
| PPM1B | protein phosphatase, Mg2+/Mn2+ dependent 1B |
| PPP1R15B | protein phosphatase 1 regulatory subunit 15B |
| PQLC3 | solute carrier family 66 member 3 |
| PRDX3 | peroxiredoxin 3 |
| PREB | prolactin regulatory element binding |
| PTCD3 | pentatricopeptide repeat domain 3 |
| PTGER3 | prostaglandin E receptor 3 |
| PTRF | caveolae associated protein 1 |
| QDPR | quinoid dihydropteridine reductase |
| RAB34 | RAB34, member RAS oncogene family |
| REEP5 | receptor accessory protein 5 |
| REEP6 | receptor accessory protein 6 |
| RETN | resistin |
| RETSAT | retinol saturase |
| RIOK3 | RIO kinase 3 |
| RNF11 | ring finger protein 11 |
| RREB1 | ras responsive element binding protein 1 |
| RTN3 | reticulon 3 |
| SAMM50 | SAMM50 sorting and assembly machinery component |
| SCARB1 | scavenger receptor class B member 1 |
| SCP2 | sterol carrier protein 2 |
| SDHB | succinate dehydrogenase complex iron sulfur subunit B |
| SDHC | succinate dehydrogenase complex iron sulfur subunit C |
| SDPR | caveolae associated protein 2 |
| SLC19A1 | solute carrier family 19 member 1 |
| SLC1A5 | solute carrier family 1 member 5 |
| SLC25A1 | solute carrier family 25 member 1 |
| SLC25A10 | solute carrier family 25 member 10 |
| SLC27A1 | solute carrier family 27 member 1 |
| SLC5A6 | solute carrier family 5 member 6 |
| SNCG | synuclein gamma |
| SOD1 | superoxide dismutase 1 |
| SORBS1 | sorbin and SH3 domain containing 1 |
| SPARCL1 | SPARC like 1 |
| SQRDL | sulfide quinone oxidoreductase |
| SSPN | sarcospan |
| STAT5A | signal transducer and activator of transcription 5A |
| STOM | stomatin |
| SUCLG1 | succinate-CoA ligase GDP/ADP-forming subunit alpha |
| SULT1A1 | sulfotransferase family 1A member 1 |
| TALDO1 | transaldolase 1 |
| TANK | TRAF family member associated NFKB activator |
| TKT | transketolase |
| TOB1 | transducer of ERBB2, 1 |
| TST | thiosulfate sulfurtransferase |
| UBC | ubiquitin C |
| UBQLN1 | ubiquilin 1 |
| UCK1 | uridine-cytidine kinase 1 |
| UCP2 | uncoupling protein 2 |
| UQCR10 | ubiquinol-cytochrome c reductase, complex III subunit X |
| UQCR11 | ubiquinol-cytochrome c reductase, complex III subunit XI |
| UQCRC1 | ubiquinol-cytochrome c reductase core protein 1 |
| UQCRQ | ubiquinol-cytochrome c reductase complex III subunit VII |
| VEGFB | vascular endothelial growth factor B |
| YWHAG | tyrosine 3-monooxygenase/tryptophan 5-monooxygenase activation protein gamma |

Table S2. Comparison of clinical and pathological features between low and high adipogenesis score with breast cancer in the GSE96058 cohort.

| GSE96058 |  | Low | High |  |
| --- | --- | --- | --- | --- |
| Characteristics |  | (*n* = 1636) | (*n* = 1637) | *p*-value |
| Age | Median | 62 | 66 | < 0.001 |
|  | IQR | 51-70 | 56-73 |  |
| Subtype | ER+/HER2- | 1125 | 1300 | < 0.001 |
|  | TNBC | 103 | 40 |  |
|  | HER2+ | 221 | 199 |  |
|  | Unknown | 187 | 98 |  |
| AJCC |  |  |  |  |
| T-category | T0 | 204 | 212 | 0.644 |
|  | T1 | 853 | 843 |  |
|  | T2 | 500 | 496 |  |
|  | T3 | 41 | 30 |  |
|  | T4 | 6 | 9 |  |
|  | Unknown | 42 | 37 |  |
| N-category | N- | 1365 | 1379 | 0.687 |
|  | N+ | 232 | 225 |  |
|  | Unknown | 39 | 33 |  |
| M-category | M- | 1605 | 1609 | 1.00 |
|  | M+ | 1 | 2 |  |
|  | Unknown | 30 | 26 |  |
| Nottingham Grade | 1 | 265 | 231 | < 0.001 |
|  | 2 | 707 | 825 |  |
|  | 3 | 633 | 551 |  |
|  | Unknown | 31 | 30 |  |

AJCC, American Joint Committee of Cancer; ER, estrogen receptor; HER2, human epidermal growth factor receptor 2; IQR, interquartile range; TNBC, triple negative breast cancer

Table S3. Comparison of clinical and pathological features between low and high adipogenesis score with breast cancer in the TCGA cohort.

| TCGA |  | Low | High |  |
| --- | --- | --- | --- | --- |
| Characteristics |  | (*n* = 534) | (*n* = 535) | *p*-value |
| Age | Median |  |  |  |
|  | IQR |  |  |  |
| Subtype | ER+/HER2- | 271 | 310 | < 0.001 |
|  | TNBC | 105 | 54 |  |
|  | HER2+ | 74 | 102 |  |
|  | Unknown | 84 | 69 |  |
| AJCC |  |  |  |  |
| Stage | I | 86 | 92 | 0.073 |
|  | II | 321 | 283 |  |
|  | III | 107 | 136 |  |
|  | IV | 7 | 11 |  |
|  | Unknown | 13 | 13 |  |
| T-category | T1 | 135 | 138 | 0.475 |
|  | T2 | 318 | 298 |  |
|  | T3 | 63 | 74 |  |
|  | T4 | 16 | 22 |  |
|  | Unknown | 2 | 3 |  |
| N-category | N- | 260 | 246 | 0.665 |
|  | N+ | 270 | 271 |  |
|  | Unknown | 4 | 18 |  |
| M-category | M- | 454 | 433 | 0.371 |
|  | M+ | 8 | 12 |  |
|  | Unknown | 72 | 90 |  |
| Nottingham Grade | 1 | 40 | 36 | 0.615 |
|  | 2 | 129 | 133 |  |
|  | 3 | 124 | 107 |  |
|  | Unknown | 241 | 259 |  |

AJCC, American Joint Committee of Cancer; ER, estrogen receptor; HER2, human epidermal growth factor receptor 2; IQR, interquartile range; TNBC, triple negative breast cancer

**Table S4: Association of individual genes in the adipogenesis score with TNBC patient survival in both GSE96058 and TCGA cohorts. *P*-value was calculated using log-rank test.**

|  | GSE96058 (OS) | | | | | TCGA (DSS) | | | | | TCGA (OS) | | | | |
| --- | --- | --- | --- | --- | --- | --- | --- | --- | --- | --- | --- | --- | --- | --- | --- |
| Gene | HR | 95%CI | | | *p* | HR | 95%CI | | | *p* | HR | 95%CI | | | *p* |
| ABCA1 | 0.85 | 0.58 | - | 1.25 | 0.413 | 1.18 | 0.71 | - | 1.96 | 0.525 | 1.14 | 0.75 | - | 1.71 | 0.541 |
| ABCB8 | 1.51 | 0.88 | - | 2.56 | 0.131 | 2.74 | 1.32 | - | 5.70 | 0.007 | 2.26 | 1.25 | - | 4.08 | 0.007 |
| ACAA2 | 0.88 | 0.59 | - | 1.30 | 0.511 | 0.63 | 0.35 | - | 1.13 | 0.122 | 0.59 | 0.36 | - | 0.97 | 0.039 |
| ACADL | 1.05 | 0.88 | - | 1.26 | 0.557 | 0.91 | 0.79 | - | 1.04 | 0.158 | 0.97 | 0.87 | - | 1.08 | 0.565 |
| ACADM | 0.86 | 0.59 | - | 1.25 | 0.422 | 0.49 | 0.27 | - | 0.88 | 0.016 | 0.67 | 0.40 | - | 1.11 | 0.117 |
| ACADS | 1.02 | 0.63 | - | 1.66 | 0.936 | 1.26 | 0.72 | - | 2.21 | 0.421 | 1.21 | 0.77 | - | 1.90 | 0.413 |
| ACLY | 1.19 | 0.59 | - | 2.39 | 0.635 | 1.06 | 0.43 | - | 2.62 | 0.897 | 1.20 | 0.55 | - | 2.60 | 0.644 |
| ACO2 | 2.08 | 1.17 | - | 3.68 | 0.012 | 0.51 | 0.20 | - | 1.29 | 0.155 | 0.88 | 0.43 | - | 1.80 | 0.727 |
| ACOX1 | 0.93 | 0.52 | - | 1.68 | 0.820 | 0.27 | 0.11 | - | 0.71 | 0.007 | 0.40 | 0.19 | - | 0.86 | 0.018 |
| ADCY6 | 1.42 | 0.88 | - | 2.31 | 0.155 | 1.25 | 0.66 | - | 2.37 | 0.495 | 1.95 | 1.13 | - | 3.38 | 0.017 |
| ADIG | 2.65 | 0.62 | - | #### | 0.188 | 1.31 | 0.95 | - | 1.79 | 0.096 | 1.20 | 0.90 | - | 1.59 | 0.212 |
| ADIPOQ | 1.01 | 0.86 | - | 1.19 | 0.900 | 0.97 | 0.88 | - | 1.08 | 0.616 | 1.01 | 0.93 | - | 1.10 | 0.803 |
| ADIPOR2 | 0.76 | 0.44 | - | 1.31 | 0.318 | 0.54 | 0.21 | - | 1.40 | 0.206 | 0.36 | 0.16 | - | 0.82 | 0.015 |
| AGPAT3 | 0.45 | 0.24 | - | 0.82 | 0.009 | 1.40 | 0.53 | - | 3.66 | 0.499 | 1.40 | 0.64 | - | 3.06 | 0.402 |
| AIFM1 | 1.27 | 0.76 | - | 2.12 | 0.369 | 1.25 | 0.58 | - | 2.66 | 0.569 | 1.33 | 0.71 | - | 2.51 | 0.375 |
| AK2 | 1.43 | 0.79 | - | 2.59 | 0.241 | 1.27 | 0.59 | - | 2.74 | 0.543 | 1.17 | 0.62 | - | 2.22 | 0.629 |
| ALDH2 | 1.17 | 0.86 | - | 1.59 | 0.322 | 1.02 | 0.65 | - | 1.61 | 0.919 | 1.03 | 0.72 | - | 1.47 | 0.867 |
| ALDOA | 1.54 | 0.92 | - | 2.59 | 0.101 | 1.72 | 0.91 | - | 3.26 | 0.093 | 1.84 | 1.08 | - | 3.13 | 0.025 |
| ANGPT1 | 0.82 | 0.63 | - | 1.05 | 0.121 | 1.07 | 0.82 | - | 1.39 | 0.628 | 1.04 | 0.85 | - | 1.29 | 0.683 |
| ANGPTL4 | 1.17 | 0.95 | - | 1.43 | 0.132 | 1.15 | 0.89 | - | 1.47 | 0.289 | 1.14 | 0.93 | - | 1.40 | 0.209 |
| APLP2 | 1.21 | 0.78 | - | 1.86 | 0.401 | 1.06 | 0.53 | - | 2.11 | 0.869 | 1.44 | 0.83 | - | 2.47 | 0.191 |
| APOE | 0.90 | 0.67 | - | 1.22 | 0.504 | 1.22 | 0.85 | - | 1.75 | 0.279 | 1.19 | 0.89 | - | 1.60 | 0.244 |
| ARAF | 1.55 | 0.57 | - | 4.23 | 0.394 | 1.52 | 0.58 | - | 4.02 | 0.396 | 1.62 | 0.71 | - | 3.67 | 0.251 |
| ARL4A | 0.92 | 0.64 | - | 1.32 | 0.634 | 1.22 | 0.81 | - | 1.83 | 0.342 | 1.37 | 1.00 | - | 1.87 | 0.048 |
| ATL2 | 0.80 | 0.58 | - | 1.11 | 0.187 | 0.96 | 0.56 | - | 1.66 | 0.889 | 0.76 | 0.49 | - | 1.18 | 0.217 |
| ATP1B3 | 1.05 | 0.72 | - | 1.53 | 0.800 | 0.83 | 0.40 | - | 1.72 | 0.622 | 0.87 | 0.47 | - | 1.59 | 0.643 |
| BAZ2A | 1.03 | 0.43 | - | 2.45 | 0.954 | 0.49 | 0.22 | - | 1.10 | 0.084 | 0.84 | 0.42 | - | 1.69 | 0.623 |
| BCKDHA | 1.45 | 0.78 | - | 2.68 | 0.241 | 1.48 | 0.74 | - | 2.96 | 0.263 | 1.67 | 0.95 | - | 2.91 | 0.073 |
| BCL2L13 | 2.13 | 0.96 | - | 4.73 | 0.062 | 0.44 | 0.15 | - | 1.27 | 0.128 | 0.60 | 0.27 | - | 1.35 | 0.215 |
| BCL6 | 0.98 | 0.65 | - | 1.47 | 0.914 | 0.75 | 0.46 | - | 1.25 | 0.272 | 0.86 | 0.56 | - | 1.32 | 0.481 |
| C3 | 0.89 | 0.69 | - | 1.15 | 0.378 | 0.82 | 0.66 | - | 1.01 | 0.063 | 0.90 | 0.74 | - | 1.10 | 0.312 |
| CAT | 0.79 | 0.46 | - | 1.33 | 0.374 | 0.75 | 0.39 | - | 1.44 | 0.385 | 0.81 | 0.51 | - | 1.29 | 0.377 |
| CCNG2 | 0.94 | 0.57 | - | 1.54 | 0.793 | 0.60 | 0.33 | - | 1.08 | 0.090 | 0.61 | 0.37 | - | 0.99 | 0.046 |
| CD151 | 1.63 | 0.99 | - | 2.70 | 0.055 | 2.78 | 1.51 | - | 5.10 | 0.001 | 2.35 | 1.43 | - | 3.85 | 0.001 |
| CD302 | 0.89 | 0.61 | - | 1.30 | 0.554 | 0.78 | 0.49 | - | 1.23 | 0.280 | 0.86 | 0.59 | - | 1.26 | 0.432 |
| CD36 | 1.00 | 0.82 | - | 1.23 | 0.993 | 1.10 | 0.86 | - | 1.40 | 0.443 | 1.15 | 0.93 | - | 1.41 | 0.189 |
| CDKN2C | 0.91 | 0.63 | - | 1.32 | 0.634 | 0.91 | 0.60 | - | 1.38 | 0.655 | 0.86 | 0.62 | - | 1.20 | 0.370 |
| CHCHD10 | 1.77 | 1.08 | - | 2.91 | 0.023 | 1.09 | 0.72 | - | 1.64 | 0.682 | 1.08 | 0.76 | - | 1.52 | 0.678 |
| CHUK | 1.17 | 0.56 | - | 2.48 | 0.676 | 0.62 | 0.30 | - | 1.29 | 0.200 | 0.75 | 0.40 | - | 1.40 | 0.359 |
| CIDEA | 1.00 | 0.86 | - | 1.16 | 0.997 | 0.99 | 0.87 | - | 1.13 | 0.904 | 1.03 | 0.93 | - | 1.14 | 0.579 |
| CMBL | 1.01 | 0.80 | - | 1.28 | 0.905 | 1.06 | 0.82 | - | 1.37 | 0.682 | 1.07 | 0.86 | - | 1.32 | 0.546 |
| CMPK1 | 0.93 | 0.39 | - | 2.19 | 0.865 | 0.76 | 0.28 | - | 2.07 | 0.595 | 0.67 | 0.29 | - | 1.56 | 0.354 |
| COL15A1 | 1.01 | 0.71 | - | 1.44 | 0.950 | 0.80 | 0.54 | - | 1.21 | 0.294 | 0.99 | 0.69 | - | 1.42 | 0.954 |
| COL4A1 | 1.08 | 0.73 | - | 1.61 | 0.701 | 0.99 | 0.58 | - | 1.69 | 0.976 | 1.07 | 0.69 | - | 1.65 | 0.757 |
| COQ3 | 0.99 | 0.67 | - | 1.46 | 0.950 | 0.85 | 0.48 | - | 1.47 | 0.554 | 0.65 | 0.41 | - | 1.02 | 0.059 |
| COQ5 | 0.94 | 0.51 | - | 1.74 | 0.844 | 1.75 | 0.60 | - | 5.08 | 0.304 | 1.52 | 0.64 | - | 3.60 | 0.342 |
| COQ9 | 1.48 | 0.85 | - | 2.58 | 0.171 | 0.78 | 0.36 | - | 1.71 | 0.540 | 0.84 | 0.44 | - | 1.62 | 0.609 |
| COX6A1 | 1.06 | 0.63 | - | 1.79 | 0.821 | 1.33 | 0.70 | - | 2.53 | 0.377 | 1.31 | 0.78 | - | 2.20 | 0.311 |
| COX7B | 1.11 | 0.67 | - | 1.84 | 0.691 | 1.49 | 0.76 | - | 2.90 | 0.243 | 1.28 | 0.73 | - | 2.24 | 0.383 |
| COX8A | 1.36 | 0.77 | - | 2.39 | 0.289 | 1.72 | 0.92 | - | 3.24 | 0.092 | 1.47 | 0.87 | - | 2.50 | 0.150 |
| CPT2 | 1.46 | 0.86 | - | 2.48 | 0.164 | 1.24 | 0.47 | - | 3.28 | 0.665 | 2.00 | 0.89 | - | 4.53 | 0.095 |
| CRAT | 1.04 | 0.86 | - | 1.27 | 0.674 | 1.05 | 0.78 | - | 1.41 | 0.767 | 1.22 | 0.97 | - | 1.53 | 0.091 |
| CS | 2.39 | 1.10 | - | 5.18 | 0.028 | 0.60 | 0.21 | - | 1.74 | 0.350 | 0.97 | 0.43 | - | 2.18 | 0.947 |
| CYC1 | 1.34 | 0.89 | - | 2.03 | 0.159 | 2.29 | 1.34 | - | 3.89 | 0.002 | 2.01 | 1.28 | - | 3.16 | 0.002 |
| CYP4B1 | 0.99 | 0.83 | - | 1.19 | 0.944 | 0.98 | 0.84 | - | 1.14 | 0.748 | 1.05 | 0.93 | - | 1.19 | 0.414 |
| DBT | 0.95 | 0.51 | - | 1.78 | 0.874 | 0.49 | 0.19 | - | 1.22 | 0.124 | 0.65 | 0.30 | - | 1.39 | 0.265 |
| DDT | 1.86 | 1.15 | - | 2.99 | 0.011 | 0.80 | 0.44 | - | 1.44 | 0.456 | 0.94 | 0.59 | - | 1.52 | 0.813 |
| DECR1 | 0.96 | 0.53 | - | 1.73 | 0.895 | 2.02 | 0.96 | - | 4.22 | 0.062 | 1.34 | 0.73 | - | 2.47 | 0.343 |
| DGAT1 | 1.68 | 1.05 | - | 2.69 | 0.029 | 3.05 | 1.68 | - | 5.53 | 0.000 | 2.62 | 1.56 | - | 4.39 | 0.000 |
| DHCR7 | 1.34 | 0.99 | - | 1.81 | 0.061 | 0.90 | 0.56 | - | 1.43 | 0.653 | 1.05 | 0.72 | - | 1.53 | 0.799 |
| DHRS7 | 1.34 | 0.83 | - | 2.15 | 0.227 | 1.22 | 0.68 | - | 2.21 | 0.503 | 1.21 | 0.75 | - | 1.95 | 0.444 |
| DHRS7B | 1.67 | 0.99 | - | 2.82 | 0.054 | 1.44 | 0.79 | - | 2.63 | 0.239 | 1.54 | 0.94 | - | 2.53 | 0.090 |
| DLAT | 1.57 | 0.86 | - | 2.88 | 0.140 | 0.99 | 0.43 | - | 2.26 | 0.980 | 1.12 | 0.57 | - | 2.23 | 0.742 |
| DLD | 1.30 | 0.66 | - | 2.58 | 0.447 | 0.84 | 0.36 | - | 1.93 | 0.676 | 1.14 | 0.61 | - | 2.14 | 0.677 |
| DNAJB9 | 0.87 | 0.49 | - | 1.52 | 0.619 | 0.97 | 0.47 | - | 2.00 | 0.932 | 1.17 | 0.66 | - | 2.09 | 0.590 |
| DNAJC15 | 1.20 | 0.86 | - | 1.69 | 0.279 | 1.02 | 0.62 | - | 1.69 | 0.935 | 1.16 | 0.73 | - | 1.83 | 0.534 |
| DRAM2 | 1.11 | 0.55 | - | 2.23 | 0.769 | 1.26 | 0.49 | - | 3.28 | 0.634 | 0.95 | 0.44 | - | 2.01 | 0.885 |
| ECH1 | 1.06 | 0.71 | - | 1.56 | 0.788 | 1.51 | 0.81 | - | 2.83 | 0.198 | 1.60 | 0.98 | - | 2.62 | 0.063 |
| ECHS1 | 1.17 | 0.65 | - | 2.11 | 0.603 | 0.92 | 0.47 | - | 1.79 | 0.806 | 1.01 | 0.59 | - | 1.74 | 0.959 |
| ELMOD3 | 1.22 | 0.73 | - | 2.04 | 0.445 | 2.03 | 0.92 | - | 4.47 | 0.081 | 2.18 | 1.14 | - | 4.14 | 0.018 |
| ELOVL6 | 1.05 | 0.78 | - | 1.41 | 0.754 | 1.13 | 0.77 | - | 1.67 | 0.536 | 1.00 | 0.72 | - | 1.38 | 0.996 |
| ENPP2 | 0.74 | 0.57 | - | 0.97 | 0.029 | 0.92 | 0.68 | - | 1.24 | 0.576 | 0.99 | 0.78 | - | 1.25 | 0.934 |
| EPHX2 | 1.14 | 0.85 | - | 1.53 | 0.371 | 1.06 | 0.68 | - | 1.65 | 0.808 | 1.42 | 0.93 | - | 2.17 | 0.105 |
| ESRRA | 1.74 | 0.95 | - | 3.19 | 0.071 | 1.80 | 1.01 | - | 3.21 | 0.048 | 1.54 | 0.96 | - | 2.46 | 0.074 |
| ESYT1 | 1.67 | 0.75 | - | 3.71 | 0.206 | 0.71 | 0.28 | - | 1.80 | 0.475 | 0.82 | 0.39 | - | 1.73 | 0.599 |
| ETFB | 1.64 | 1.00 | - | 2.70 | 0.052 | 1.61 | 1.05 | - | 2.48 | 0.031 | 1.40 | 1.01 | - | 1.95 | 0.044 |
| FABP4 | 1.00 | 0.88 | - | 1.13 | 0.956 | 1.09 | 0.95 | - | 1.25 | 0.243 | 1.09 | 0.98 | - | 1.22 | 0.113 |
| FAH | 1.19 | 0.94 | - | 1.51 | 0.143 | 1.08 | 0.66 | - | 1.78 | 0.751 | 1.17 | 0.79 | - | 1.73 | 0.424 |
| FZD4 | 0.99 | 0.72 | - | 1.35 | 0.947 | 0.89 | 0.58 | - | 1.37 | 0.599 | 1.06 | 0.74 | - | 1.50 | 0.755 |
| G3BP2 | 1.39 | 0.77 | - | 2.48 | 0.272 | 0.74 | 0.37 | - | 1.50 | 0.407 | 0.75 | 0.43 | - | 1.31 | 0.308 |
| GADD45A | 1.07 | 0.73 | - | 1.56 | 0.730 | 1.04 | 0.61 | - | 1.77 | 0.898 | 0.81 | 0.53 | - | 1.25 | 0.338 |
| GBE1 | 1.45 | 0.90 | - | 2.34 | 0.125 | 1.02 | 0.57 | - | 1.82 | 0.959 | 1.03 | 0.62 | - | 1.70 | 0.921 |
| GHITM | 1.23 | 0.59 | - | 2.56 | 0.578 | 0.50 | 0.17 | - | 1.49 | 0.215 | 1.05 | 0.47 | - | 2.33 | 0.909 |
| GPAM | 0.95 | 0.67 | - | 1.34 | 0.759 | 0.82 | 0.46 | - | 1.45 | 0.498 | 0.79 | 0.49 | - | 1.27 | 0.325 |
| GPD2 | 0.96 | 0.65 | - | 1.42 | 0.838 | 0.41 | 0.23 | - | 0.74 | 0.003 | 0.51 | 0.30 | - | 0.87 | 0.013 |
| GPHN | 1.61 | 0.96 | - | 2.70 | 0.073 | 0.96 | 0.58 | - | 1.60 | 0.877 | 1.32 | 0.84 | - | 2.05 | 0.226 |
| GPX3 | 0.93 | 0.68 | - | 1.27 | 0.664 | 1.05 | 0.74 | - | 1.51 | 0.773 | 1.11 | 0.83 | - | 1.48 | 0.490 |
| GPX4 | 1.67 | 1.00 | - | 2.80 | 0.051 | 1.46 | 0.85 | - | 2.51 | 0.167 | 1.32 | 0.85 | - | 2.06 | 0.217 |
| GRPEL1 | 1.41 | 0.83 | - | 2.39 | 0.203 | 1.68 | 0.77 | - | 3.67 | 0.197 | 1.93 | 1.02 | - | 3.63 | 0.042 |
| HADH | 1.25 | 0.80 | - | 1.97 | 0.330 | 1.56 | 0.84 | - | 2.88 | 0.156 | 1.64 | 1.00 | - | 2.71 | 0.052 |
| HIBCH | 1.71 | 1.15 | - | 2.54 | 0.008 | 0.57 | 0.27 | - | 1.21 | 0.143 | 0.98 | 0.59 | - | 1.64 | 0.948 |
| HSPB8 | 1.07 | 0.85 | - | 1.36 | 0.566 | 1.13 | 0.88 | - | 1.44 | 0.342 | 1.16 | 0.95 | - | 1.41 | 0.149 |
| IDH1 | 1.20 | 0.85 | - | 1.69 | 0.305 | 0.55 | 0.29 | - | 1.04 | 0.066 | 0.68 | 0.40 | - | 1.17 | 0.163 |
| IDH3A | 1.55 | 0.81 | - | 2.99 | 0.189 | 1.30 | 0.54 | - | 3.11 | 0.555 | 1.28 | 0.63 | - | 2.61 | 0.495 |
| IDH3G | 1.02 | 0.49 | - | 2.13 | 0.961 | 1.63 | 0.82 | - | 3.24 | 0.166 | 1.37 | 0.77 | - | 2.44 | 0.287 |
| IFNGR1 | 0.81 | 0.44 | - | 1.49 | 0.503 | 0.84 | 0.45 | - | 1.56 | 0.577 | 0.86 | 0.51 | - | 1.43 | 0.555 |
| IMMT | 2.34 | 1.08 | - | 5.08 | 0.031 | 0.65 | 0.21 | - | 2.02 | 0.459 | 0.86 | 0.35 | - | 2.11 | 0.743 |
| ITGA7 | 0.92 | 0.70 | - | 1.20 | 0.549 | 1.24 | 0.90 | - | 1.69 | 0.187 | 1.19 | 0.92 | - | 1.54 | 0.185 |
| ITIH5 | 0.89 | 0.70 | - | 1.12 | 0.320 | 1.08 | 0.82 | - | 1.41 | 0.585 | 1.10 | 0.88 | - | 1.38 | 0.395 |
| ITSN1 | 0.84 | 0.45 | - | 1.55 | 0.576 | 0.81 | 0.40 | - | 1.63 | 0.551 | 0.83 | 0.46 | - | 1.50 | 0.542 |
| JAGN1 | 0.83 | 0.45 | - | 1.51 | 0.536 | 1.42 | 0.69 | - | 2.96 | 0.343 | 1.13 | 0.61 | - | 2.10 | 0.699 |
| LAMA4 | 1.10 | 0.74 | - | 1.65 | 0.635 | 1.20 | 0.74 | - | 1.93 | 0.464 | 1.39 | 0.94 | - | 2.07 | 0.101 |
| LEP | 0.94 | 0.76 | - | 1.15 | 0.537 | 0.97 | 0.84 | - | 1.12 | 0.668 | 0.99 | 0.89 | - | 1.11 | 0.881 |
| LIFR | 0.85 | 0.65 | - | 1.10 | 0.209 | 0.83 | 0.62 | - | 1.12 | 0.223 | 0.85 | 0.68 | - | 1.07 | 0.170 |
| LIPE | 0.96 | 0.76 | - | 1.21 | 0.735 | 1.10 | 0.85 | - | 1.42 | 0.455 | 1.09 | 0.89 | - | 1.35 | 0.404 |
| LPCAT3 | 1.12 | 0.81 | - | 1.54 | 0.498 | 0.76 | 0.39 | - | 1.48 | 0.417 | 0.89 | 0.51 | - | 1.55 | 0.683 |
| LPL | 0.92 | 0.76 | - | 1.12 | 0.428 | 0.93 | 0.75 | - | 1.17 | 0.544 | 1.03 | 0.86 | - | 1.24 | 0.714 |
| LTC4S | 1.13 | 0.79 | - | 1.61 | 0.513 | 1.23 | 0.95 | - | 1.60 | 0.115 | 1.26 | 1.02 | - | 1.57 | 0.031 |
| MAP4K3 | 0.69 | 0.34 | - | 1.41 | 0.309 | 0.64 | 0.30 | - | 1.35 | 0.238 | 0.68 | 0.36 | - | 1.28 | 0.232 |
| MCCC1 | 0.79 | 0.54 | - | 1.17 | 0.236 | 0.66 | 0.36 | - | 1.21 | 0.180 | 0.76 | 0.48 | - | 1.22 | 0.254 |
| MDH2 | 2.47 | 1.20 | - | 5.10 | 0.015 | 1.23 | 0.53 | - | 2.88 | 0.628 | 1.41 | 0.70 | - | 2.83 | 0.332 |
| ME1 | 0.92 | 0.68 | - | 1.24 | 0.586 | 0.81 | 0.54 | - | 1.22 | 0.320 | 0.77 | 0.55 | - | 1.07 | 0.118 |
| MGLL | 1.30 | 0.92 | - | 1.83 | 0.140 | 1.12 | 0.74 | - | 1.70 | 0.578 | 1.35 | 0.97 | - | 1.88 | 0.077 |
| MGST3 | 1.02 | 0.64 | - | 1.61 | 0.948 | 1.21 | 0.59 | - | 2.48 | 0.609 | 0.81 | 0.45 | - | 1.47 | 0.490 |
| MRAP | 0.96 | 0.72 | - | 1.29 | 0.797 | 1.01 | 0.89 | - | 1.15 | 0.831 | 1.04 | 0.94 | - | 1.15 | 0.493 |
| MRPL15 | 1.06 | 0.68 | - | 1.64 | 0.803 | 2.31 | 1.25 | - | 4.28 | 0.008 | 1.43 | 0.87 | - | 2.33 | 0.156 |
| MTCH2 | 1.56 | 0.86 | - | 2.84 | 0.142 | 0.70 | 0.29 | - | 1.69 | 0.424 | 0.64 | 0.32 | - | 1.25 | 0.187 |
| MYLK | 0.87 | 0.69 | - | 1.11 | 0.265 | 0.86 | 0.63 | - | 1.17 | 0.343 | 0.94 | 0.73 | - | 1.21 | 0.634 |
| NABP1 | 0.63 | 0.39 | - | 1.02 | 0.062 | . | . | - | . | . | . | . | - | . | . |
| NDUFA5 | 1.04 | 0.56 | - | 1.95 | 0.900 | 0.93 | 0.43 | - | 2.01 | 0.848 | 0.93 | 0.51 | - | 1.72 | 0.827 |
| NDUFAB1 | 0.82 | 0.44 | - | 1.52 | 0.531 | 2.39 | 0.97 | - | 5.90 | 0.059 | 2.04 | 0.97 | - | 4.27 | 0.059 |
| NDUFB7 | 1.65 | 0.96 | - | 2.84 | 0.069 | 1.33 | 0.81 | - | 2.17 | 0.260 | 1.21 | 0.80 | - | 1.83 | 0.362 |
| NDUFS3 | 1.07 | 0.52 | - | 2.20 | 0.845 | 1.04 | 0.49 | - | 2.20 | 0.927 | 0.79 | 0.43 | - | 1.47 | 0.459 |
| NKIRAS1 | 0.85 | 0.44 | - | 1.67 | 0.640 | 1.01 | 0.40 | - | 2.55 | 0.981 | 0.84 | 0.39 | - | 1.81 | 0.651 |
| NMT1 | 0.62 | 0.26 | - | 1.52 | 0.299 | 1.32 | 0.35 | - | 4.92 | 0.681 | 1.06 | 0.36 | - | 3.11 | 0.920 |
| OMD | 0.95 | 0.78 | - | 1.16 | 0.629 | 0.81 | 0.64 | - | 1.01 | 0.066 | 0.96 | 0.79 | - | 1.17 | 0.695 |
| ORM1 | 1.05 | 0.94 | - | 1.17 | 0.395 | 1.02 | 0.92 | - | 1.13 | 0.696 | 1.03 | 0.94 | - | 1.12 | 0.518 |
| PDCD4 | 0.99 | 0.67 | - | 1.47 | 0.975 | 0.99 | 0.61 | - | 1.62 | 0.973 | 1.14 | 0.77 | - | 1.70 | 0.516 |
| PEMT | 0.96 | 0.57 | - | 1.61 | 0.867 | 1.87 | 1.05 | - | 3.33 | 0.034 | 1.38 | 0.85 | - | 2.26 | 0.193 |
| PEX14 | 1.05 | 0.64 | - | 1.72 | 0.841 | 1.93 | 0.91 | - | 4.09 | 0.085 | 1.75 | 0.95 | - | 3.21 | 0.072 |
| PFKFB3 | 1.10 | 0.70 | - | 1.75 | 0.671 | 1.22 | 0.74 | - | 2.02 | 0.428 | 1.19 | 0.79 | - | 1.78 | 0.410 |
| PFKL | 2.13 | 1.09 | - | 4.14 | 0.026 | 2.83 | 1.33 | - | 6.02 | 0.007 | 2.24 | 1.21 | - | 4.16 | 0.011 |
| PGM1 | 0.85 | 0.55 | - | 1.31 | 0.456 | 1.06 | 0.61 | - | 1.83 | 0.838 | 1.06 | 0.67 | - | 1.67 | 0.808 |
| PHLDB1 | 1.09 | 0.70 | - | 1.71 | 0.702 | 1.65 | 0.86 | - | 3.16 | 0.132 | 1.23 | 0.75 | - | 2.00 | 0.415 |
| PHYH | 0.88 | 0.59 | - | 1.32 | 0.542 | 0.90 | 0.57 | - | 1.43 | 0.657 | 0.82 | 0.57 | - | 1.16 | 0.259 |
| PIM3 | 1.09 | 0.68 | - | 1.74 | 0.716 | 0.82 | 0.46 | - | 1.48 | 0.512 | 0.86 | 0.53 | - | 1.40 | 0.538 |
| PLIN2 | 1.40 | 0.98 | - | 1.99 | 0.064 | 1.15 | 0.63 | - | 2.10 | 0.648 | 1.02 | 0.62 | - | 1.68 | 0.938 |
| POR | 1.62 | 1.02 | - | 2.56 | 0.040 | 1.24 | 0.66 | - | 2.31 | 0.506 | 1.44 | 0.86 | - | 2.42 | 0.163 |
| PPARG | 1.13 | 0.86 | - | 1.48 | 0.378 | 1.03 | 0.69 | - | 1.54 | 0.898 | 1.08 | 0.78 | - | 1.49 | 0.640 |
| PPM1B | 0.94 | 0.41 | - | 2.16 | 0.881 | 0.30 | 0.14 | - | 0.65 | 0.002 | 0.28 | 0.14 | - | 0.54 | 0.000 |
| PPP1R15B | 0.84 | 0.40 | - | 1.78 | 0.648 | 0.43 | 0.15 | - | 1.27 | 0.129 | 0.45 | 0.18 | - | 1.12 | 0.087 |
| PQLC3 | 0.80 | 0.48 | - | 1.31 | 0.370 | 0.87 | 0.48 | - | 1.57 | 0.640 | 1.04 | 0.61 | - | 1.78 | 0.886 |
| PRDX3 | 1.16 | 0.65 | - | 2.06 | 0.621 | 0.49 | 0.20 | - | 1.18 | 0.111 | 0.68 | 0.33 | - | 1.42 | 0.308 |
| PREB | 1.06 | 0.48 | - | 2.33 | 0.880 | 3.01 | 1.00 | - | 9.09 | 0.051 | 1.42 | 0.58 | - | 3.48 | 0.447 |
| PTCD3 | 0.92 | 0.52 | - | 1.64 | 0.790 | 0.66 | 0.24 | - | 1.82 | 0.422 | 0.79 | 0.36 | - | 1.71 | 0.541 |
| PTGER3 | 0.74 | 0.56 | - | 0.98 | 0.034 | 1.05 | 0.81 | - | 1.35 | 0.733 | 1.08 | 0.87 | - | 1.33 | 0.497 |
| QDPR | 1.63 | 0.96 | - | 2.78 | 0.071 | 1.58 | 0.77 | - | 3.25 | 0.216 | 1.68 | 0.95 | - | 2.96 | 0.072 |
| RAB34 | 0.77 | 0.52 | - | 1.13 | 0.179 | 1.63 | 0.84 | - | 3.14 | 0.148 | 1.01 | 0.62 | - | 1.65 | 0.955 |
| REEP5 | 1.20 | 0.86 | - | 1.67 | 0.292 | 1.38 | 0.77 | - | 2.48 | 0.276 | 1.85 | 1.17 | - | 2.91 | 0.008 |
| REEP6 | 1.11 | 0.98 | - | 1.25 | 0.104 | 1.08 | 0.91 | - | 1.29 | 0.388 | 1.13 | 0.98 | - | 1.30 | 0.094 |
| RETN | 1.10 | 0.90 | - | 1.34 | 0.370 | 1.15 | 0.98 | - | 1.34 | 0.082 | 1.04 | 0.92 | - | 1.18 | 0.499 |
| RETSAT | 1.30 | 0.97 | - | 1.75 | 0.079 | 0.86 | 0.47 | - | 1.57 | 0.617 | 1.36 | 0.85 | - | 2.18 | 0.204 |
| RIOK3 | 0.88 | 0.49 | - | 1.57 | 0.660 | 0.58 | 0.26 | - | 1.26 | 0.169 | 0.60 | 0.33 | - | 1.11 | 0.105 |
| RMDN3 | 0.82 | 0.46 | - | 1.49 | 0.521 | . | . | - | . | . | . | . | - | . | . |
| RNF11 | 1.07 | 0.57 | - | 2.01 | 0.836 | 1.01 | 0.46 | - | 2.24 | 0.977 | 0.99 | 0.52 | - | 1.89 | 0.980 |
| RREB1 | 1.47 | 0.80 | - | 2.70 | 0.217 | 0.58 | 0.33 | - | 1.04 | 0.068 | 0.81 | 0.49 | - | 1.33 | 0.403 |
| RTN3 | 1.95 | 1.07 | - | 3.57 | 0.030 | 0.39 | 0.16 | - | 0.95 | 0.037 | 0.55 | 0.27 | - | 1.12 | 0.100 |
| SAMM50 | 2.52 | 1.16 | - | 5.45 | 0.019 | 1.78 | 0.62 | - | 5.12 | 0.288 | 2.03 | 0.84 | - | 4.88 | 0.115 |
| SCARB1 | 0.89 | 0.66 | - | 1.19 | 0.417 | 0.79 | 0.53 | - | 1.18 | 0.250 | 0.76 | 0.55 | - | 1.07 | 0.112 |
| SCP2 | 1.07 | 0.76 | - | 1.52 | 0.697 | 0.62 | 0.25 | - | 1.50 | 0.285 | 0.89 | 0.43 | - | 1.82 | 0.750 |
| SDHB | 1.56 | 0.75 | - | 3.23 | 0.237 | 0.73 | 0.28 | - | 1.88 | 0.514 | 0.86 | 0.44 | - | 1.68 | 0.654 |
| SDHC | 0.84 | 0.46 | - | 1.52 | 0.559 | 1.21 | 0.50 | - | 2.92 | 0.666 | 1.40 | 0.67 | - | 2.93 | 0.365 |
| SLC19A1 | 0.88 | 0.56 | - | 1.37 | 0.577 | 2.34 | 1.35 | - | 4.06 | 0.003 | 1.61 | 1.05 | - | 2.49 | 0.031 |
| SLC1A5 | 1.06 | 0.67 | - | 1.68 | 0.808 | 1.31 | 0.66 | - | 2.59 | 0.437 | 1.56 | 0.89 | - | 2.75 | 0.121 |
| SLC25A1 | 1.58 | 0.91 | - | 2.72 | 0.102 | 1.66 | 0.92 | - | 3.00 | 0.090 | 1.58 | 0.97 | - | 2.60 | 0.069 |
| SLC25A10 | 1.08 | 0.77 | - | 1.51 | 0.657 | 0.90 | 0.53 | - | 1.54 | 0.697 | 0.92 | 0.60 | - | 1.43 | 0.720 |
| SLC27A1 | 1.05 | 0.66 | - | 1.66 | 0.847 | 0.95 | 0.50 | - | 1.78 | 0.867 | 0.92 | 0.57 | - | 1.47 | 0.728 |
| SLC5A6 | 1.01 | 0.76 | - | 1.36 | 0.928 | 1.26 | 0.79 | - | 2.01 | 0.323 | 1.08 | 0.75 | - | 1.56 | 0.671 |
| SNCG | 0.89 | 0.73 | - | 1.09 | 0.272 | 1.16 | 0.95 | - | 1.44 | 0.152 | 1.08 | 0.92 | - | 1.27 | 0.367 |
| SOD1 | 0.76 | 0.42 | - | 1.37 | 0.369 | 1.41 | 0.72 | - | 2.76 | 0.310 | 1.15 | 0.66 | - | 2.01 | 0.612 |
| SORBS1 | 0.83 | 0.62 | - | 1.12 | 0.232 | 0.67 | 0.47 | - | 0.95 | 0.027 | 0.71 | 0.52 | - | 0.96 | 0.026 |
| SOWAHC | 1.05 | 0.65 | - | 1.70 | 0.848 | . | . | - | . | . | . | . | - | . | . |
| SPARCL1 | 0.77 | 0.55 | - | 1.07 | 0.117 | 1.06 | 0.75 | - | 1.49 | 0.737 | 1.12 | 0.85 | - | 1.48 | 0.424 |
| SSPN | 0.91 | 0.67 | - | 1.23 | 0.535 | 0.63 | 0.41 | - | 0.98 | 0.042 | 0.68 | 0.47 | - | 0.98 | 0.039 |
| STAT5A | 0.78 | 0.52 | - | 1.16 | 0.220 | 1.08 | 0.58 | - | 2.01 | 0.813 | 1.02 | 0.60 | - | 1.72 | 0.952 |
| STOM | 0.74 | 0.46 | - | 1.19 | 0.211 | 0.91 | 0.48 | - | 1.74 | 0.785 | 1.41 | 0.81 | - | 2.46 | 0.223 |
| SUCLG1 | 1.34 | 0.67 | - | 2.66 | 0.407 | 1.40 | 0.52 | - | 3.75 | 0.503 | 1.47 | 0.66 | - | 3.26 | 0.346 |
| SULT1A1 | 1.00 | 0.70 | - | 1.44 | 0.979 | 1.13 | 0.73 | - | 1.76 | 0.588 | 1.26 | 0.87 | - | 1.83 | 0.214 |
| TALDO1 | 1.44 | 0.79 | - | 2.60 | 0.231 | 2.36 | 1.16 | - | 4.81 | 0.018 | 1.56 | 0.89 | - | 2.76 | 0.124 |
| TANK | 0.53 | 0.30 | - | 0.95 | 0.032 | 0.40 | 0.19 | - | 0.87 | 0.020 | 0.45 | 0.24 | - | 0.83 | 0.011 |
| TKT | 1.47 | 0.93 | - | 2.35 | 0.102 | 1.65 | 0.94 | - | 2.89 | 0.082 | 1.57 | 0.98 | - | 2.51 | 0.064 |
| TOB1 | 1.16 | 0.80 | - | 1.68 | 0.435 | 0.76 | 0.45 | - | 1.26 | 0.287 | 0.94 | 0.61 | - | 1.46 | 0.787 |
| TST | 1.65 | 1.12 | - | 2.42 | 0.010 | 1.22 | 0.81 | - | 1.84 | 0.336 | 1.15 | 0.84 | - | 1.56 | 0.389 |
| UBC | 1.61 | 0.78 | - | 3.34 | 0.202 | 1.37 | 0.52 | - | 3.60 | 0.518 | 1.40 | 0.64 | - | 3.10 | 0.401 |
| UBQLN1 | 0.59 | 0.26 | - | 1.31 | 0.191 | 0.70 | 0.27 | - | 1.82 | 0.466 | 0.97 | 0.47 | - | 2.02 | 0.945 |
| UCK1 | 0.73 | 0.35 | - | 1.52 | 0.405 | 2.45 | 0.94 | - | 6.39 | 0.066 | 2.08 | 1.01 | - | 4.28 | 0.048 |
| UCP2 | 1.01 | 0.74 | - | 1.38 | 0.969 | 0.99 | 0.64 | - | 1.52 | 0.959 | 1.08 | 0.76 | - | 1.54 | 0.649 |
| UQCR10 | 1.29 | 0.69 | - | 2.41 | 0.423 | 1.21 | 0.62 | - | 2.38 | 0.571 | 1.28 | 0.75 | - | 2.18 | 0.363 |
| UQCR11 | 1.21 | 0.63 | - | 2.31 | 0.562 | 1.84 | 1.08 | - | 3.16 | 0.026 | 1.65 | 1.05 | - | 2.61 | 0.031 |
| UQCRC1 | 1.60 | 1.12 | - | 2.28 | 0.010 | 1.60 | 0.78 | - | 3.27 | 0.201 | 1.58 | 0.86 | - | 2.92 | 0.141 |
| UQCRQ | 1.11 | 0.66 | - | 1.88 | 0.693 | 1.61 | 0.96 | - | 2.69 | 0.070 | 1.66 | 1.08 | - | 2.56 | 0.021 |
| VEGFB | 1.79 | 0.88 | - | 3.65 | 0.107 | 1.39 | 0.63 | - | 3.04 | 0.416 | 1.29 | 0.69 | - | 2.43 | 0.420 |
| YWHAG | 1.88 | 0.92 | - | 3.87 | 0.085 | 0.71 | 0.26 | - | 1.89 | 0.490 | 0.67 | 0.31 | - | 1.45 | 0.313 |

CI, Confidence Interval; HR, hazard ratio.

**Table S5. Genes used to calculate each immune cell, including CD8+, CD4+ memory, T helper type 1 and type 2 cell, Tregs, and M1 and M2 macrophage.**

**CD8^+^ T cell:**

AAK1, APBB1, ARHGEF1, BTN2A1, C7orf26, CA6, CASP8, CBY1, CCDC25, CCDC53, CCR7, CD160, CD27, CD3D, CD7, CD8A, CD8B, CD96, CEPT1, CIAPIN1, CLUAP1, COG2, COPZ1, CRTAM, CTSW, CX3CR1, DHX15, DIDO1, DNAJB1, DPP8, DSC1, EEF1D, EML3, FAM134C, FBXW4, FKTN, FNBP4, FTO, GGNBP2, GIMAP4, GJC2, GZMH, GZMK, GZMM, HNRNPA0, HNRNPL, IL16, IPCEF1, IRF3, KLHL3, KLRB1, KLRG1, KRT2, LAIR2, LSM14A, LY9, MED17, MKRN2, MMP19, MSL3, MTRF1, MYOM1, NAA16, NDFIP1, NDUFS2, NFKB1, NKRF, NPAT, NPRL2, PCNT, PFN2, PLCG1, PLXDC1, POLR3E, POP5, PRL, PRMT2, PRPF4B, PSD, PTGDR, PTPN4, PURA, RAPGEF6, RASA2, RBL2, RBM34, RING1, RNF113A, RPL37A, RWDD3, S100B, SDAD1, SDCCAG3, SFPQ, SHANK1, SIRPG, SLC1A7, SSTR3, TBCC, TMEM41B, TOMM7, TRAF3IP3, TSPAN32, TTN, UBE2Q1, UBQLN2, USP47, UTP20, WDR82, YLPM1, ZBTB11, ZC3HAV1, ZNF154, ZNF200, ZNF611, ZNF639

**CD4^+^ memory T cell:**

AAMP, ACD, ACTL6A, ADSL, AHCTF1, AKT2, AMBRA1, ANP32B, ANXA7, API5, ARHGAP15, ARL2, ARPC4, ATF1, ATG5, ATPIF1, ATXN10, AURKAIP1, BAG3, BCAS2, BTF3, BUB3, C11orf58, C12orf29, CBLL1, CBX3, CCNC, CCR4, CD2, CD226, CD28, CD2AP, CD3G, CD40LG, CD5, CD6, CD96, CDC40, CDK9, CDKN2AIP, CDV3, CEP57, CETN3, CLPX, CMPK1, CNBP, COPS4, COPS5, COX7C, CPSF6, CSNK1A1, CSNK2A2, CTBP1, CTLA4, CXCR6, DAD1, DAP3, DBF4, DDX3X, DDX50, DENR, DLEC1, DNAJA2, DNAJB1, DOHH, DPM1, DR1, EEF2, EID1, EIF2B5, EIF3E, EIF3L, EIF3M, EIF4G2, ERH, ESD, ETAA1, EXOC2, FARS2, FCF1, FNTA, FUBP3, FXR1, GABPA, GALR2, GATAD2A, GLOD4, GLUD1, GPR132, GPR15, GPR171, GPR183, GRPEL1, GZMA, GZMK, HDAC1, HINT1, HMGB2, HMGN4, HMOX2, HNRNPA0, HNRNPH3, HNRNPU, ICOS, IMP3, INTS8, ISCA1, ITK, JAK3, KARS, KBTBD4, KIF22, KTN1, LDHB, LIMS1, LIN7C, MAEA, MAGOH, MATR3, MEN1, METAP1, METTL5, MMADHC, MRPL11, MRPL20, MRPL44, MRPS18B, MRPS27, MRPS34, NAE1, NCL, NDUFS5, NKRF, NUPL2, SGEP, PABPC4, PAPOLA, PCID2, PCNP, PDCD1, PDCD10, PFDN6, PFN1, PKP4, PLP2, POLD2, PPA2, PPID, PPIH, PPP1CB, PPP1CC, PPP2R5D, PPP6C, PREPL, PRPF18, PRPF19, PSMF1, PTGES3, PTPN11, PTPN4, RAD21, RANBP1, RANBP9, RBL2, RBM3, RBM34, RGS1, RNF34, RNF6, RPF1, RPL13, RPL13A, RPL36, RPL4, RPL5, RPL8, RPS19, RPS3, RPS6, RRP1B, RSL24D1, RUVBL1, RWDD1, SAFB2, SEC23IP, SERP1, SH2D1A, SLAMF1, SLC25A38, SLC25A6, SMAD2, SMC5, SMU1, SOD1, SP3, SPAG16, SRP9, SSNA1, STK16, SUB1, SUCLG1, SURF2, TBL3, THAP11, THOC7, THOP1, THRAP3, TINF2, TPP2, TPT1, TRA2A, TRAT1, TRMT112, TSN, TSSC1, TTC37, U2AF2, UBASH3A, UBE2D2, UBE2D3, UBE2N, UBIAD1, UBQLN2, UNC45A, UQCRC2, USP39, UXT, WDR46, ZC3H15, ZDHHC6, ZNF236, ZZZ3

**T helper type 1 cell:**

TH1, CDC123, CHD1L, CHD4, COX10, CSTF1, CUEDC2, EIF2B2, FIBP, GNLY, HTRA2, IFNG, KIF20A, LAG3, MDC1, MNAT1, NCAPD3, NUP205, PKMYT1, POLD2, PPM1G, PSMD3, PTTG1, R3HDM1, RNPS1, RUVBL2, SLAMF1, SNRPC, TACO1, THOP1, TMEM39B, TRIM28, TTLL5, UBAP2, WDR18, WRAP53, ZBTB32

**T helper type 2 cell:**

GZMK, IL5, IL13, MAD2L1, RRM2, BAG2, CXCR6, CEP55, RRAS2, NUP37, NPHP4, GPR15, GZMA, SMAD2, CDK2AP1, RGS9, SLC25A44, RAD50, TMEM39B, UBAP2, THADA, RNF34

**Tregs:**

ATG2B, BANP, CCR3, CCR4, CCR8, CD28, CD5, CTLA4, CXCR6, FOXP3, GALNT8, GPR25, HS3ST3B1, ICOS, IKZF4, IL10RA, IL2RA, IPCEF1, ITGB7, KCNA2, LAIR2, LAX1, LRP2BP, MCF2L2, MCM9, PLCL1, PPM1B, RGS1, SIT1, SPTAN1, STAM, TTN, TULP4, UBE4A, VPS54, ZCCHC8, ZFC3H1, ZMYM1, ZNF236

**M1 macrophage:**

ABCD1, ABI1, ABTB2, ACP2, ACTR2, ACTR3, ADAMDEC1, ADCK2, ADCY3, ADO, ADRA2B, AFG3L2, AGPS, ALCAM, ANXA2, AP1M2, ARHGEF11, ARL8B, ATOX1, ATP6V0C, ATP6V1A, ATP6V1D, ATP6V1E1, ATP6V1F, ATP6V1H, BCAP31, BCKDK, BLVRA, C1QA, C1QB, C3AR1, CALR, CCDC47, CCL1, CCL18, CCL19, CCL22, CCL24, CCL7, CCL8, CCR1, CD163, CD300C, CD48, CD63, CD80, CD84, CECR5, CHIT1, CIAO1, CLCN7, CLEC4E, CLPB, CLTC, CMKLR1, COQ2, CORO7, COX5B, CSF1, CSF1R, CXCL9, CYBB, CYC1, CYFIP1, CYP19A1, DAGLA, DLAT, DNAJC13, DNASE2B, DOT1L, EMILIN1, EXOC5, FAM32A, FANCE, FCER1G, FDX1, FKBP15, FOLR2, FPR2, FPR3, FTL, GLRX2, GP1BA, GPD1, HAMP, HAUS2, HEXB, HK3, HSPB7, HYAL2, IFNAR1, IGSF6, IL10, IL12B, IL17RA, ITGAE, ITGB1BP1, KCNJ1, KCNJ5, KCNK13, KIFC3, LAIR1, LAMP1, LILRB1, LILRB4, LIMD2, LONP1, LONRF3, MAPK13, MARCO, MFSD7, MMP19, MRPL12, MRPL40, MRS2, MS4A4A, MSR1, MT2A, MYBPH, MYH11, MYO7A, MYOF, MYOZ1, NARS, NCAPH, NDUFAF1, NDUFS2, NECAP2, NRBP1, OGFR, OTUD4, P2RX7, PDCL, PHLDB1, PKD2L1, PLEKHB2, PQLC2, PRDX1, PTGIR, PTPRA, RAB3IL1, RELA, RNH1, RRP1, S100A11, S1PR2, SCAMP2, SDS, SIGLEC1, SIGLEC7, SIGLEC9, SLAMF8, SLC11A1, SLC1A2, SLC25A24, SLC31A1, SLC6A12, SNX3, SPG21, SPR, SRC, STIP1, STX12, STX4, TBC1D16, TCEB1, TDRD7, TFEC, TFRC, TIE1, TMEM33, TMEM70, TMX1, TPP1, TREM2, TRIP4, TSPO, UQCR11, USP14, UTP3, VIM, VPS33A, VSIG4, WDR11, WSB2, WTAP, ZC3H15, ZMPSTE24

**M2 macrophage:**

ABCD1, ACP2, ACSM5, ADAMDEC1, ADCY3, ADRA2B, AGGF1, AKR7A2, ALDH9A1, ALG9, ALK, ANGPT4, ANKFY1, ANXA11, AP1B1, AQP8, ARFGEF2, ARHGEF11, ARSB, ATP2A2, ATP6V0A1, ATP6V0D1, ATP6V1C1, ATP6V1D, BAIAP2, BCAP31, BTBD1, C10orf76, C16orf62, CAMP, CANX, CARD14, CCDC85C, CCDC88A, CD52, CD63, CD81, CDS2, CEPT1, CLCN7, COL4A3BP, COMMD9, CYFIP1, DHX57, DNASE1L3, DNASE2B, EFR3A, ELK1, EXOC1, FDX1, FGR, FH, FKBP15, FLT1, FTL, GABARAP, GGA1, GLB1, GORASP1, GPD1, GSTO1, GUCA1A, HADHB, HAMP, HEXA, HEXB, HPS1, HS3ST2, HSPH1, IARS2, IFNAR1, IPPK, ITGAX, KCNJ1, KCNJ5, KCNK13, KCTD5, KIAA0196, LAIR1, LAMP1, LILRA2, LILRB4, LONRF3, MARCO, MFN1, MMP19, MRM1, MS4A4A, MSR1, MTMR14, MYO15A, MYO9B, MYOZ1, NAGPA, NCAPH, NCKAP1L, NDUFB1, NFS1, NOP10, NPR1, OS9, OSBPL11, P2RX7, PABPC4, PDCD6IP, PDE1B, PEX19, PICK1, PLEKHM2, POGK, PQLC2, RIN2, S100A6, SCAMP2, SDCBP, SDS, SLAMF8, SLC25A24, SLC25A46, SLC31A1, SLC38A7, SLC39A1, SLC6A12, SLC6A7, SLC9A6, SMG5, SNAPC2, SNX1, SNX2, SNX3, SNX5, SPG21, STX18, STX4, TAF10, TBC1D9B, TFEC, TMED5, TMEM184C, TMEM70, TMEM9B, TNFSF14, TPP1, TREM2, TSPO, UBXN6, UCP3, UGP2, UNC50, USF2, VPS35, VPS53, VSIG4, VTI1B, WDFY3, XPNPEP2, ZC3H3, ZCCHC4, ZNF219


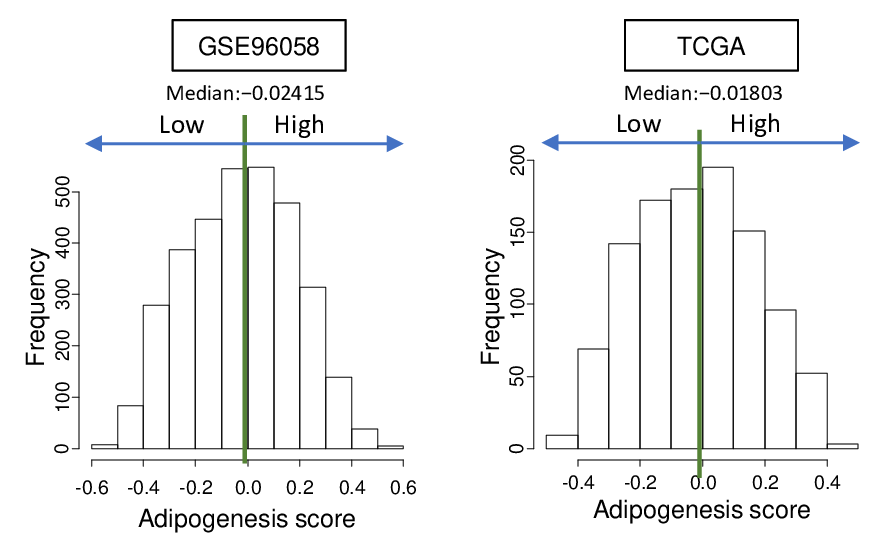


**Figure S1: Histogram of adipogenesis score and number of participants in each group of the GSE96058 and TCGA cohorts.**


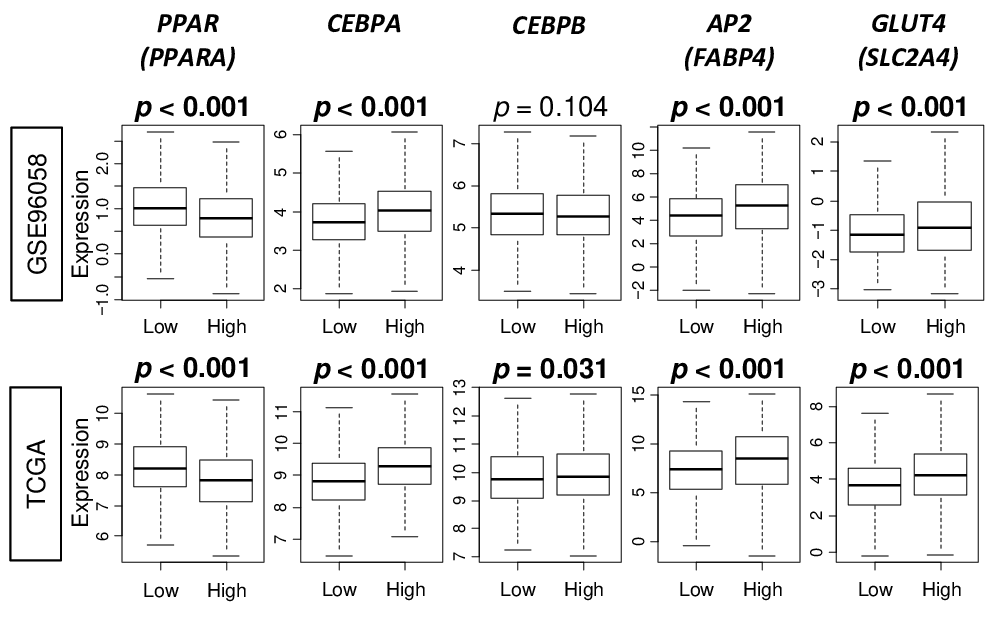


**Figure S2.** **Association of the adipogenesis score with expression of adipogenesis-related genes in the GSE96058 and TCGA cohort.** Boxplots comparing low and high adipogenesis score tumors of gene expression levels of *PPAR* (*PPARA*)*, CEBPA, CEBPB, AP2 (FABP4),* and *GLUT4 (SLC2A4)* in breast cancer. Median value was used as the cut-off to divide into low and high adipogenesis score groups within each cohort. *P*-value was calculated using Mann-Whitney U test.

**
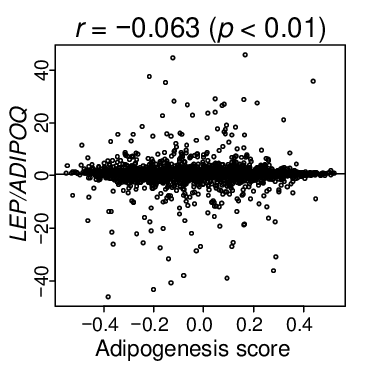
**

**Figure S3: Association of the adipogenesis score with leptin (*LEP*) to adiponectin (*ADIPOQ*) in the GSE96058 cohort.** Correlation plots of the adipogenesis score with LEP to ADIPOQ expression ratios in the GSE96058 cohort. Spearman correlation statistics was used in the analysis.

**
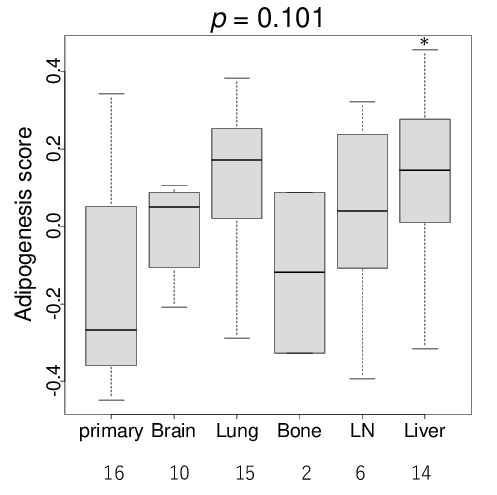
**

**Figure S4: Association of the adipogenesis score with metastatic breast cancer in the GSE110590 cohort.** Boxplots of the adipogenesis score by each breast tumor, including primary site, brain, lung, bone, lymph node, and liver metastasis. *P*-value was calculated using Kruskal-Wallis test. * show the significant difference compared to primary breast cancer by Mann-Whitney U test.

**
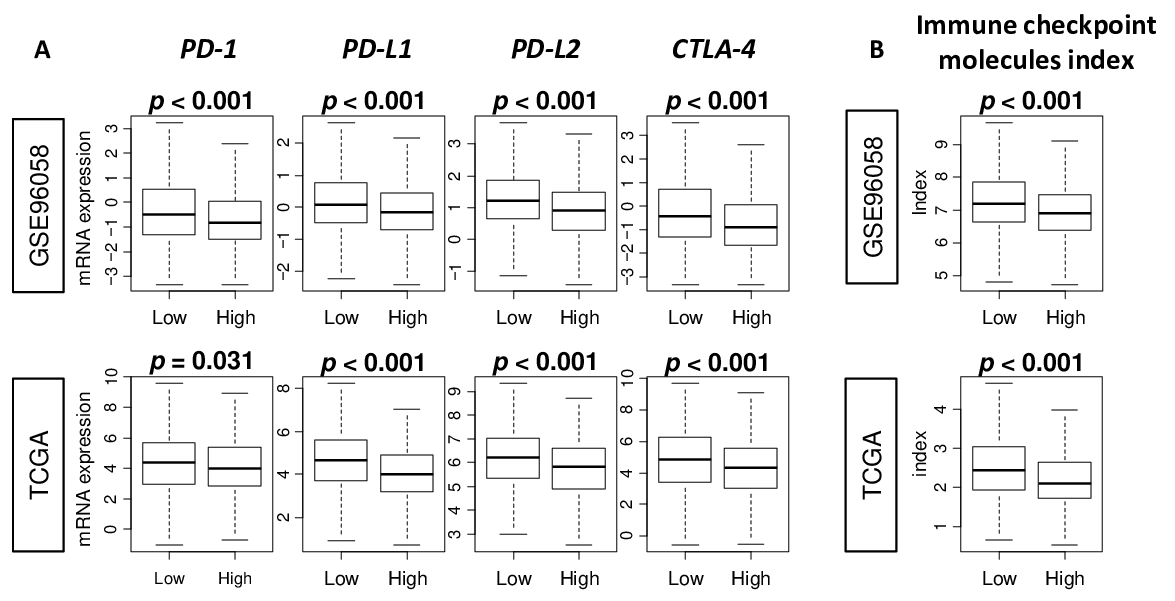
**

**Figure S5. Association of the adipogenesis score with expression of immune checkpoint molecules in whole breast cancer cohort.** **(A)** Boxplots of comparison between low- and high-adipogenesis score with mRNA expression of PD-1/*PDCD1*, PD-L1/*CD274*, PD-L2/*PDCD1LG2*, and *CTLA4* genes, and **(B)** immune checkpoint molecules index in whole breast cancer in the GSE96058 and TCGA cohorts. *P*-value was calculated using Mann-Whitney U test. Median value was used as the cut-off to divide into low- and high- adipogenesis score groups within each cohort. PD-1; programmed cell death 1; PD-L1; programmed cell death 1 ligand 1, PD-L2; programmed cell death 1 ligand 2, CTLA4; cytotoxic T-lymphocyte-associated protein 4.
